# Natural killer cells and IFN-γ protect against liver injury during HAV infection in mice

**DOI:** 10.1128/jvi.01395-25

**Published:** 2025-09-19

**Authors:** Ichiro Misumi, You Li, Takayoshi Shirasaki, Lixin Yang, Maryna Kapustina, Stanley M. Lemon, Jason K. Whitmire

**Affiliations:** 1 Department of Genetics, The University of North Carolina at Chapel Hill 2331, Chapel Hill, North Carolina, USA; 2 Department of Pediatrics, The University of North Carolina at Chapel Hill 2331, Chapel Hill, North Carolina, USA; 3 Lineberger Comprehensive Cancer Center, The University of North Carolina at Chapel Hill 2331, Chapel Hill, North Carolina, USA; 4 Department of Cell Biology & Physiology, The University of North Carolina at Chapel Hill 2331, Chapel Hill, North Carolina, USA; 5 Department of Medicine, The University of North Carolina at Chapel Hill 2331, Chapel Hill, North Carolina, USA; 6 Department of Microbiology & Immunology, The University of North Carolina at Chapel Hill 2331, Chapel Hill, North Carolina, USA; Wake Forest University School of Medicine, Winston-Salem, North Carolina, USA

**Keywords:** HAV, hepatitis A, NK cells, T cells, interferons, mouse models

## Abstract

A limited number of studies on human subjects with type-A hepatitis have found a positive correlation between activated natural killer (NK) cell frequencies in blood and the severity of liver injury. However, without knowledge of differences in viral burdens, it remains unclear whether NK cells are elevated due to hepatitis A virus (HAV) replication and ongoing associated injury, or if NK cell responses directly cause pathogenesis. To gain insight into how NK cells respond during the early stages of hepatitis A virus infection, we generated mice that are permissive for HAV infection in the liver but are otherwise immunocompetent. HAV readily infected the liver of these hepatocyte-specific type-I interferon receptor knockout mice (*Ifnar1^ΔHep^*), resulting in fecal virus shedding, elevated serum alanine aminotransferase levels, and immune cell infiltrates in the liver. Single-cell RNA sequencing analyses of liver leukocytes from infected *Ifnar1^ΔHep^* mice revealed a rapid accumulation of activated NK cells in the liver within 2 days. HAV infection and liver inflammation were brief and substantially reduced in magnitude in these mice compared to global *Ifnar1^-/-^* knockout mice that failed to induce substantial antiviral NK cell responses. NK cells were the principal source of interferon (IFN)-γ in *Ifnar1^ΔHep^* mice. *Ifnar1^ΔHep^* mice that were experimentally depleted of NK cells or IFN-γ showed greatly increased viral replication and liver damage. Moreover, IFN-γ alone was sufficient to suppress HAV replication in primary mouse hepatocytes. Collectively, these findings establish a critical role for type-I IFN induction of NK cells, their expression of IFN-γ, and protection against hepatitis A.

**IMPORTANCE:**

Hepatitis A virus remains a leading cause of foodborne illness among unvaccinated individuals. Infection can result in severe liver injury that can progress to fatal fulminant viral hepatitis. Despite its clinical significance, the molecular, genetic, and cellular factors influencing infection severity remain poorly understood. Previous studies of patient blood have suggested that natural killer (NK) cells cause liver injury during infection. In this study, we used mouse models of infection to characterize early cellular defenses to infection and identified a critical role for NK cells and interferon-γ in conferring rapid immune protection rather than pathogenesis.

## INTRODUCTION

Hepatitis A virus (HAV) is a positive-sense, single-stranded RNA virus classified within the *Hepatovirus* genus of the *Picornaviridae* family (1, 2). It is remarkably stable in the environment and typically transmitted via the fecal-oral route. Once acquired, it is strictly tropic for hepatocytes where it replicates, producing quasi-enveloped virions that can be found in blood and naked particles that are shed in feces (3, 4). Despite the availability of effective vaccines, HAV remains a global health concern among unvaccinated individuals (5). Sporadic outbreaks in the United States have been linked to contaminated food and person-to-person transmission, possibly facilitated by the sharing of contaminated needles (6–8). While HAV infection is generally self-limiting, severe cases can progress to fulminant hepatitis with liver failure, particularly among older adults and patients with chronic hepatitis B virus or hepatitis C virus (HCV) infections (9–13). The mechanisms underlying these diverse clinical outcomes remain poorly understood. However, recently developed mouse models of infection that recapitulate human disease provide a valuable tool to address these knowledge gaps (14).

It is unclear whether cytolytic immune cell populations contribute to HAV pathogenesis. HAV is generally considered noncytopathic because its replication in hepatocytes does not induce immediate cell lysis, and there is a longstanding notion that cytolytic CD8+ T cells mediate liver injury during infection (15). Longitudinal studies in human subjects and in non-human primate models found that peak viremia precedes peak alanine aminotransferase (ALT) activity (16), which has been interpreted as evidence that active virus replication does not cause liver injury. Moreover, viremia is reduced in subjects with fulminant hepatitis (17), which aligns with the notion that vigorous antiviral T cell responses contribute to disease severity. Contemporary approaches can now be used to track virus-specific T cells in infected people and in non-human primate models (18, 19), while distinguishing them from non-HAV-specific cells. A human study implicated IL15-stimulated bystander memory CD8+ T cell responses in liver injury during HAV infection (20, 21). These bystander cells, which were specific for other pathogens, were recruited to the liver during HAV infection, where they engaged hepatocytes via NKG2D and NKp30 receptors and triggered hepatocyte cell death. However, a longitudinal study of non-human primates that tracked virus-specific T cells over the course of infection found that virus-specific T cells emerge after peak levels of HAV shedding, viremia, and ALT activity (19). As T cell frequencies increased, there was a reduction in HAV production and ALT activity (19), consistent with a protective role for antiviral T cells that prevent infection-related liver injury. Moreover, virus-infected mice showed an inverse relationship between the magnitude of virus-specific T cells and ALT levels, consistent with T cell-mediated immune protection against viral pathogenesis (22).

The function of intrahepatic NK cells remains poorly defined, largely because available studies are limited and have drawn inferences based on peripheral blood analyses rather than direct liver sampling and quantification of viral burden (23–25). Comparisons of human subjects with type A hepatitis versus healthy controls (23) revealed a positive correlation between elevated frequencies of activated NK cells in blood versus circulating levels of ALT and aspartate transferase, which are liver-derived enzymes that are released by injured hepatocytes. A recent study of an individual who died of fulminant viral hepatitis identified genetic evidence for a homozygous loss-of-function mutation in IL-18BP (26), a factor that can be secreted by hepatocytes and macrophages to block IL-18 signaling. Among many factors, IL-18 can activate NK cells and induce cytolytic activity in NK92 cells against HAV-infected hepatocyte cell lines *in vitro* (26). However, without knowledge of viral burden and whether virus replication outpaces NK responses, it remains unclear whether the increase in NK cells resulted from anomalous HAV replication in this subject with associated tissue damage or if NK cell responses alone directly caused collateral injury in the course of targeting infected hepatocytes.

NK cells have a well-established role in antiviral defenses for many kinds of viral infections (27–29). As a sentinel population in lymphoid and non-lymphoid tissues, they are poised for rapid responses to infection and can be activated by a variety of mechanisms, including type-1 interferons (IFNs), type-2 interferon (IFN-γ), and cytokines, such as IL-2, IL-12, IL-15, and IL-18. NK cells register distressed cells through ligands that engage their activating receptors. They can also respond to cells with reduced surface expression of MHC molecules or to cells bound by antiviral antibodies. There are numerous activating and inhibitory NK cell receptors (30, 31), which are greatly amplified in allelically diverse populations and can result in functional heterogeneity among NK cells. Some NK cell receptors can physically interact with viral proteins to induce their antiviral effects (29). However, it is now appreciated that NK cell immunobiology is far more nuanced. In some virus infections in mice and in humans, NK cells can restrict antiviral CD8+ T cells, CD4+ T cells, and humoral immune responses through a number of mechanisms (29). The overall contribution of NK cells to immune defense thus appears to vary depending on the type of virus infection. The impact of NK cells during HAV infection remains unknown.

HAV fails to establish infection in wild-type mice because there is a robust early IFN and interferon-stimulated gene (ISG) response to viral replication in the liver that extinguishes infection (32). Primate studies have also revealed that HAV infection transiently induces an early hepatic IFN response that may be critical for restricting HAV replication in hepatocytes and possibly in immune cells (33, 34). The viral 3ABC and 3CD proteases, which likely accumulate as the virus replicates, target human MAVS and TRIF to inactivate further induction of IFNs and ISGs (35, 36). However, the viral protease fails to recognize murine MAVS and induces robust IFN and ISG responses in mice (32). To develop a mouse model of infection, we circumvented this process by challenging *Ifnar1^-/-^* (type-1 interferon receptor-1 knock-out) mice. HAV targets hepatocytes and replicates to high titer in *Ifnar1*^-/-^ mice, which develop hallmark features of hepatitis A, including substantial hepatic immune cell infiltration, increased ALT activity, pro-inflammatory gene expression, and hepatocyte apoptosis (37, 38). By comparing infections of *Mavs^-/-^* and *Irf3^-/-^* mice and other knockout mice, we demonstrated that most of these effects are due to MAVS-IRF3 signaling and consequent transcriptional responses that lead to hepatocyte cell death (37, 39). We previously used antibodies to deplete NK cells prior to infecting *Ifnar1^-/-^* mice and found no impact on the severity of hepatitis (37). However, it is possible that any beneficial role of NK cells in *Ifnar1*^-/-^ mice may have been obscured by the absence of IFNAR signaling and its broad antiviral effects. Moreover, it may be that IFNAR signaling is essential for NK cell responses to HAV, and eliminating unresponsive NK cells in *Ifnar1^-/-^* mice failed to affect outcomes.

Here, we have investigated NK cell responses during the early stages of HAV infection using *Albumin^Cre^;Ifnar1^fl/fl^* (*Ifnar1^ΔHep^*) mice. We show that these mice, with hepatocyte-specific lack of type I IFN signaling, are permissive for HAV infection and initially develop hepatitis and histological signs of liver injury, similar to human pathogenesis. However, compared to globally knocked-out *Ifnar1*^-/-^ mice, *Ifnar1^ΔHep^* mice rapidly controlled infection, and their liver injury quickly resolved. An enhanced immune response in *Ifnar1^ΔHep^* livers included NK cells that made cytolytic molecules, as well as IFN-γ, which directly impeded HAV replication *in vitro* and *in vivo*. These findings underscore the critical role of IFN-induced activation of NK cells and their expression of IFN-γ in preventing infection-related liver injury.

## RESULTS

### Mice lacking IFNAR1 expression only in hepatocytes are permissive for HAV infection

Wild-type (WT) mice quickly make IFNs upon challenge with HAV and are highly resistant to infection (32). In contrast, *Ifnar1^-/-^* mice sustain HAV replication because their hepatocytes cannot respond to IFN (37). However, immune defenses against HAV are also likely disabled in *Ifnar1^-/-^* mice due to the vital role IFN signaling plays in initiating immune cell responses, as observed for many virus infections. To address whether hematopoietic cell expression of IFNAR1 is critical for immune protection, we irradiated *Ifnar1^-/-^* mice and reconstituted their bone marrow with WT bone marrow cells (Fig. S1). After successfully establishing chimerism, the chimeric mice (*Ifnar1*^-/-^ with WT-BM) and unirradiated *Ifnar1^-/-^* controls were challenged intravenously with 2 × 10^6^ genome equivalents (GE) of sixth mouse-passage, mouse-adapted virus (HAV-mp6). We previously showed that a virus inoculum of this magnitude results in peak fecal shedding and liver injury at day 7 in *Ifnar1^-/-^* mice (22, 37). At day 7 post-infection (p.i.), the chimeric mice showed significantly lower serum ALT activity and reduced fecal shedding of HAV, compared to *Ifnar1^-/-^* mice (Fig. S1). These data suggest that WT *Ifnar1*^+/+^ hematopoietic cells are superior to *Ifnar1^-/-^* hematopoietic cells in suppressing HAV replication and associated liver injury. However, it is possible that WT *Ifnar1*^+/+^ immune cells established a balanced immune response that prevented excessive inflammation in the liver. Thus, to better resolve how IFN contributes to immune defense against HAV, we generated two sets of mice through intercrosses. *Ifnar1*^ΔHep^ mice (B6.*Albumin*^Cre^;*Ifnar1*^fl/fl^) selectively lack IFNAR expression in hepatocytes while preserving IFNAR expression in hematopoietic and other cell types. We also generated *Ifnar1*^ΔHepΔHsc^ mice (B6.*Albumin^Cre^;Vav^Cre^;Ifnar1^fl/fl^*) that lack IFNAR expression in hepatocytes and hematopoietic stem cell-derived cells (e.g., NK cells, myeloid cells, neutrophils, B cells, T cells, dendritic cells, etc.).

**Fig 1:**
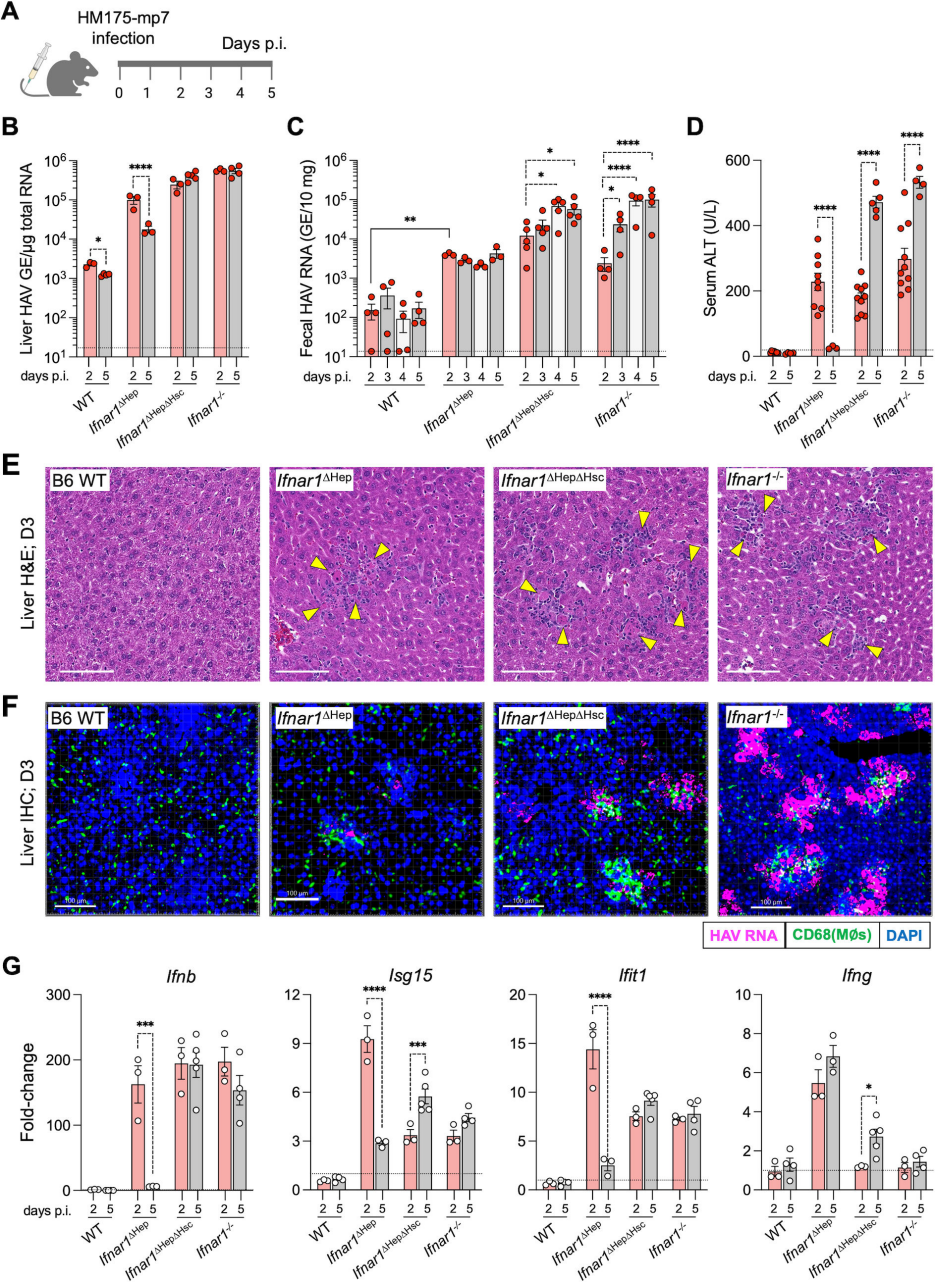
HAV infection and pathogenesis in mice with cell-type-specific loss of *Ifnar1*. (**A**) Experimental approach. Mice with the indicated genotypes were inoculated i.v. with 2 × 10^7^ GE of mouse-adapted HAV (HM175-mp7) and necropsied at days 2, 3, and 5 after infection. (**B**) Viral load in the liver on days 2 and 5 p.i. The horizontal dotted line indicates the limit of detection. (**C**) Fecal shedding of HAV from days 2 to 5 p.i. The horizontal dotted line represents the limit of detection. (**D**) Serum ALT activities. The horizontal dotted line represents activity found in uninfected WT mice. (**E**) Representative H&E-stained liver sections from mice at day 3 p.i. Yellow arrows indicate inflammatory foci and apoptotic hepatocytes; white bar = 100 µm. (**F**) Dual immunohistochemical (IHC) and RNA scope staining of CD68 (green), HAV RNA (pink), and DAPI (blue) in liver sections from infected mice; white bar = 100 μm. (**G**) qRT-qPCR determination of infection-induced changes in intrahepatic *Ifnb*, *Isg15*, *Ifit1*, and *Ifng* transcript abundance in groups of infected mice. Transcript abundance was normalized to *β-actin* mRNA in the same samples. The horizontal dotted lines indicate the amounts found in the livers of uninfected mice. The bar graphs show means ± SEM, with each dot representing individual mice. For panels **B**, **D**, and **G**, statistical testing was performed using a two-way ANOVA with Šídák’s multiple comparisons. For panel **C**, significance within each genotype was determined by two-way ANOVA with Dunnett’s test (dashed brackets) with day 2 as reference. A Mann-Whitney test was used for the pair-wise comparison between WT and *Ifnar1^ΔHep^* at day 2 (solid bracket). Significance is indicated as *P < 0.05; **P < 0.01; ***P < 0.001; ****P < 0.0001.

WT (*Cre^negative^;Ifnar1^fl/fl^*), *Ifnar1*^ΔHep^, *Ifnar1*^ΔHepΔHsc^, and *Ifnar1^-/-^* mice were challenged intravenously (i.v.) with 2 × 10^7^ GE of HAV (HM175-mp7) (16). At days 2 and 5 post-infection (p.i.), we quantified viral RNA by qRT-PCR in liver extracts, alanine aminotransferase levels in serum, a measure of liver inflammation, and intra-hepatic expression of various immune-related genes (Fig. 1A). We also quantified fecal virus shedding each day until necropsy on day 5. By day 2 post-infection, the viral RNA abundance was increased 43-fold in *Ifnar1*^ΔHep^ livers compared to livers of WT animals, consistent with productive infection (Fig. 1B). However, the amount of viral RNA in *Ifnar1*^ΔHep^ livers was less than in *Ifnar1*^ΔHepΔHsc^ and *Ifnar1^-/-^* mice. By day 5, there was a decline in viral RNA amounts in *Ifnar1*^ΔHep^ livers but not in the livers of *Ifnar1*^ΔHepΔHsc^ or *Ifnar1^-/-^* mice, suggesting that IFN-responsive hematopoietic cells exert early control of infection. As HAV replicates, new particles are released through bile into the gastrointestinal tract. At day 2, *Ifnar1*^ΔHep^ mice shed 26-fold more virus than WT mice (Fig. 1C). The shedding of virus in *Ifnar1*^ΔHep^ fecal pellets was sustained through day 5 despite the decline in viral RNA in the liver, perhaps due to the ongoing release of residual particles to the biliary tract. In contrast to *Ifnar1*^ΔHep^ mice, *Ifnar1*^ΔHepΔHsc^ and *Ifnar1^-/-^* mice showed far greater fecal virus shedding, which continued to increase through day 5, consistent with unrestricted replication in those mice.

The cohorts of mice showed substantial differences in liver injury. Serum ALT activity in *Ifnar1*^ΔHep^, *Ifnar1*^ΔHepΔHsc^, and *Ifnar1^-/-^* mice was sharply elevated at day 2 (Fig. 1D), well above the level in WT mice. However, ALT activity returned to baseline levels in *Ifnar1*^ΔHep^ mice by day 5, whereas levels in *Ifnar1*^ΔHepΔHsc^ and *Ifnar1^-/-^* mice continued to increase. The ALT patterns mirrored histological changes in the livers of infected mice (Fig. 1E; Fig. S2A). Foci containing leukocytes and apoptotic hepatocytes could be found in the livers of *Ifnar1*^ΔHep^, *Ifnar1*^ΔHepΔHsc^, and *Ifnar1^-/-^* mice at days 3 and 5, but not in WT livers. The frequency of the apoptotic hepatocytes declined in *Ifnar1*^ΔHep^ livers from days 3 to 5, mirroring the abrupt reduction in ALT in these mice; in contrast, frequencies of apoptotic hepatocytes increased from days 3 to 5 in the *Ifnar1*^ΔHepΔHsc^ and *Ifnar1^-/-^* mice. RNA-Scope *in situ* hybridization of day 3 livers revealed that viral RNA was primarily located in hepatocytes in all *Ifnar1*-deficient cohorts (Fig. 1F), confirming the hepatotropic nature of HAV in mice. CD68+ (myeloid) cells and other leukocytes were positioned adjacent to the viral RNA+ cells, especially in *Ifnar1*^ΔHepΔHsc^ and *Ifnar1^-/-^* livers, suggesting that cellular trafficking is not linked to direct IFNAR signals into leukocytes. In sum, these data show that there is a rapid and targeted accumulation of leukocytes around infected hepatocytes.

The livers of infected mice showed distinct patterns of *Ifn*, *Isg*, and *Cxcl10* expression (Fig. 1G; Fig. S2B). WT control mice (*Ifnar1^fl/fl^*, lacking Cre) effectively resisted HAV infection, likely due to the rapid induction of IFNs and ISGs at around 15–20 h p.i., as observed in C57BL/6J mice (32). As a consequence, WT livers showed minimal induction or sustained expression of these factors at day 2. *Ifnar1*^ΔHep^ livers showed transient but robust expression of *Ifnb*, *Isg15*, *Cxcl10*, *Ifit1*, and *Rsad2; Ifng* was quickly expressed and maintained to day 5. By comparison, *Ifnar1*^ΔHepΔHsc^ and *Ifnar1^-/-^* livers produced somewhat lower amounts of *Isg15*, *Ifng*, *Cxcl10*, *Ifit1*, and *Rsad2* at day 2, but sustained expression of these factors through day 5. *Ifnb* expression was similar for all three groups at day 2 but persisted to day 5 only in *Ifnar1*^ΔHepΔHsc^ and *Ifnar1^-/-^* livers. The gene expression pattern was generally similar for *Ifnar1*^ΔHepΔHsc^ and *Ifnar1^-/-^* livers, except for *Rsad2*, which increased at day 5 in *Ifnar1*^ΔHepΔHsc^ livers but not in *Ifnar1^-/-^* livers. Generally, *Ifnb*, *Cxcl10*, *Ifit1*, and *Rsad2* expression tracked with the amounts of virus in hepatocytes. At day 2, when all cohorts of mice were productively infected with comparable serum ALT elevations (Fig. 1B through D), the high levels of expression of *Isg15*, *Ifng*, *Cxcl10*, *Ifit1*, and *Rsad2* in *Ifnar1*^ΔHep^ mice imply a role for IFN-sensitive hematopoietic cells, either in directly producing these factors (e.g., *Ifng*) or supporting the expression of these factors by infected hepatocytes (perhaps *Cxcl10, Ifit1*, and *Rsad2*). The rapid expression of these factors in *Ifnar1*^ΔHep^ mice tracked with faster resolution of infection, compared to mice lacking IFNAR signaling in hematopoietic cells. In total, these findings underscore the importance of IFN signaling in hematopoietic cells to limit early HAV replication and liver damage.

### Activated NK cells rapidly accumulate in the livers of *Ifnar1*^ΔHep^ mice during HAV infection

**Fig 2:**
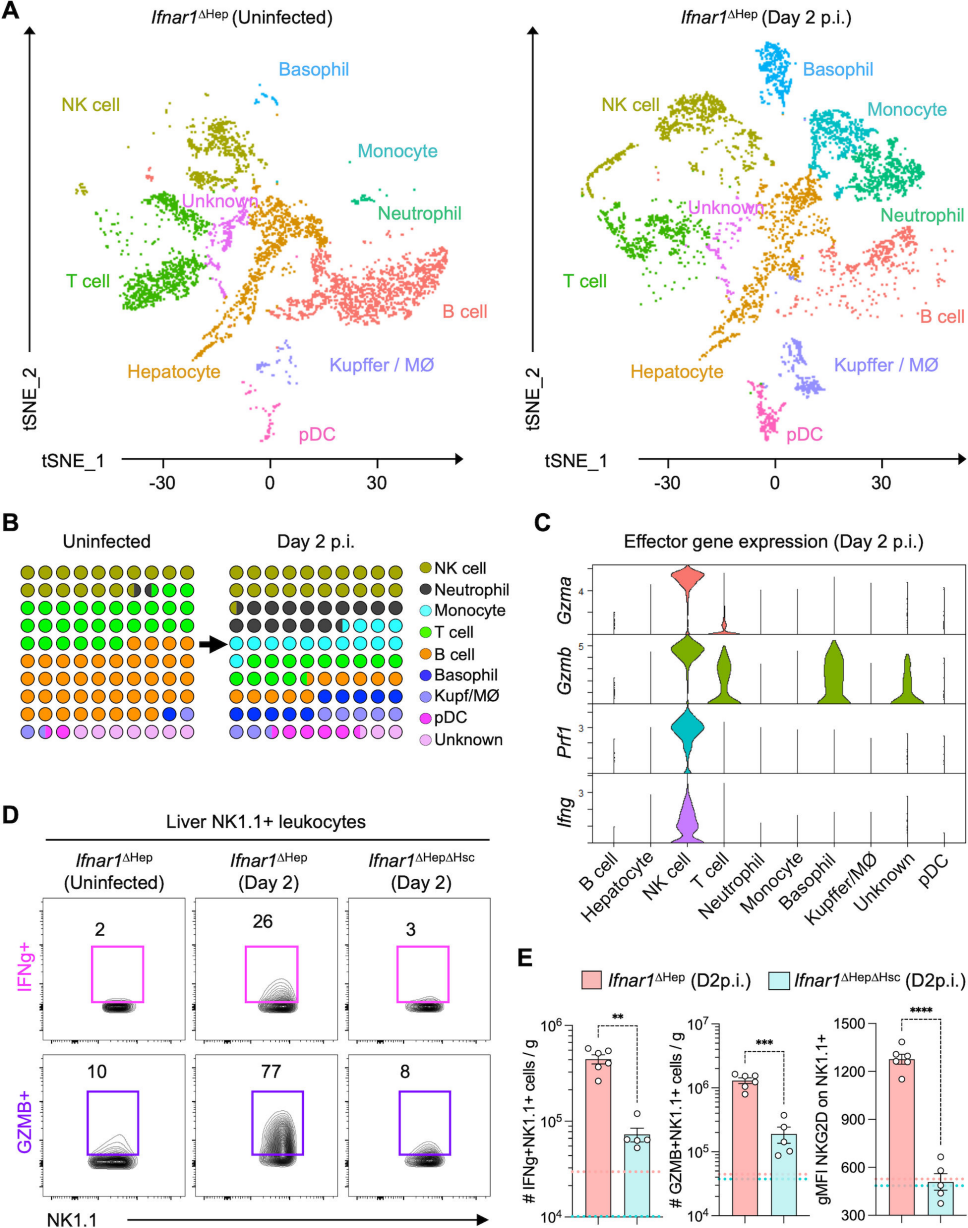
Robust NK cell responses to HAV infection in the liver of *Ifnar1*^ΔHep^ mice. Cohorts of *Ifnar1*^ΔHep^ mice were challenged intravenously with HAV infection (2 × 10^7^ GE; i.v.) or left unchallenged, and 2 days later, liver leukocytes were isolated for scRNAseq or flow cytometry analyses. (**A**) Ten major clusters of cells were resolved in the livers of mice based on compiled scRNA-seq expression data. The tSNE plots show the composition of these clusters based on their prevailing gene expression signature. The data derived from naive (*n* = 2) and HAV-infected mice (*n* = 2) are shown separately. (**B**) The 10 × 10 dot density plots depict the relative composition of CD45+ cell subsets in uninfected and day 2-infected livers. (**C**) Violin plots show the expression level of key effector genes among cell types identified in the t-SNE plots based on the scRNA-seq data of day 2 p.i. (**D**) Representative FACS plots show the percentage of NK1.1+ cells expressing IFNγ+ or granzyme B in the livers of *Ifnar1*^ΔHep^ and *Ifnar1*^ΔHepΔHsc^ mice at day 2 p.i. (*n* = 5 or 6 animals/group). The FACS profile for an uninfected *Ifnar1*^ΔHep^ mouse is shown for reference. (**E**) Bar graphs show the number of NK cells that were IFNγ+ (left) or GZMB+ (middle) per gram of liver tissue at day 2 p.i. The right bar graph shows the geometric mean fluorescence intensity of NKG2D on NK1.1+NKG2D+ cells. Each symbol represents an individual mouse. Horizontal dashed lines indicate the normal range for naive *Ifnar1*^ΔHep^ (coral) or *Ifnar1*^ΔHepΔHsc^ (turquoise). Significance was determined by Mann-Whitney test (left) or by *t*-test with Welch’s correction (middle and right) and is depicted as **P < 0.01; ***P < 0.001; and ****P < 0.0001.

The data above show that the amount of viruses in the liver was relatively similar at day 2 p.i. in the three cohorts of mice, but that infection outcomes diverged substantially afterward. We reasoned that differences in hepatic immune cell composition or immune cell gene expression could determine infection resolution or persistence. Therefore, CD45+-enriched liver cells and purified hepatocytes from two uninfected and two day 2-infected *Ifnar1*^ΔHep^ mice were subjected to single-cell RNA sequencing. This approach allowed for a comprehensive assessment of immune cell populations in the liver and their potential contributions to rapid virus control and resolution of inflammation. Gene expression patterns represented by t-distributed stochastic neighbor embedding (t-SNE) plots revealed a variety of immune cell subsets in mouse livers (Fig. 2A). The day 2 livers showed substantial increases in transcript profiles associated with NK cells, neutrophils, and other myeloid cells (Fig. 2B). Myeloid cells were prominent sources of pro-inflammatory cytokines (Fig. S3A) and chemokine signaling components (Fig. S3C and E). Neutrophils and monocytes uniquely expressed *Il1b* (Fig. S3A), although we do not know if caspase-1 activity and inflammasome activation were induced in these cells during infection. Basophils alone expressed *Il6*, a pro-inflammatory cytokine, and *Il4*, a cytokine traditionally associated with Th2 responses. The role of IL4 in viral hepatitis is unclear (40, 41). Basophil-derived IL-4 can regulate cytokine levels or contribute to inflammation during respiratory viral infections (42), and recombinant adenovirus delivery of *Il4* to the liver can trigger fatal hepatitis involving hepatocyte apoptosis, independent of immune responses (43). NK cells were the predominant source of transcripts encoding critical antiviral effector molecules (Fig. 2C), including granzymes, perforin, and IFN-γ.

CellChat analyses were used to identify plausible intercellular interactions in the liver. The analysis predicted a simplified IFNγ signaling network with IFNγR. Elevated *Ifngr1* expression in neutrophils, macrophages, and pDCs suggests a role for IFNγ in recruiting or activating myeloid cells, while hepatocytes showed no such increase in *Ifngr1* expression (Fig. S3B). NK cells also expressed high amounts of chemokines such as *Ccl3*, *Ccl4*, *Ccl5*, and *Ccr5* (Fig. S3C). Ligand-receptor pair analysis indicated that CCR signaling was likely mediated by the *Ccr1*/*Ccr5* receptors and their ligands, including *Ccl3*, *Ccl4*, and *Ccl5* (Fig. S3D). *Ccr1*-expressing neutrophils, monocytes, and basophils appear to facilitate the recruitment of Ccr5-expressing NK cells. These analyses suggest an important role for liver myeloid cells in recruiting NK cells via CCL signaling at early stages of infection. CXCR-ligand pair analysis implicated a simpler network, with major interactions driven by autocrine CXCL2-CXCR2 signaling (Fig. S3E and F). Neutrophils and monocytes were the major sources of CXCL2, which could act on CXCR2-expressing myeloid cells, including basophils. CXCL10 was expressed by various cell types, such as monocytes and macrophages, perhaps recruiting CXCR3-expressing T cells, but not NK cells.

Several distinct populations of cells in the liver can express NK1.1 (encoded by *Klrb1c*), which can be resolved by scRNAseq (Fig. S4). NK cells, which are *Klrb1^+^Tbx21*^+^*Eomes*^+^*Plzf*^neg^*Cd3*^neg^ and can be *Itga*^pos^ if liver resident, were distinguished from NK1.1^+^ ILC1 cells (*Klrb1^+^Itga1^+^Tbx21^+^Eomes*^neg^), NKT1 cells (*Klrb1^+^Cd3^+^Plzf^+^Tbx21^+^*), and activated T cells that are induced to express NK1.1^+^ (*Klrb1^+^Cd3^+^Plzf^neg^*). In uninfected livers, NK cells, ILC1, NKT cells, and T cells could all be identified using this approach (Fig. S4A and C). After infection, NK cells remained a prominent population of cells, whereas relatively few ILC1 and NKT cells were present (Fig. S4A through C). Importantly, the main cells expressing *Tbx21*, *Ifng*, and *Gzmb* after infection were authentic NK cells and some T cells; by contrast, few ILC1 or NKT cells made *Ifng* or *Gzmb* (Fig. S4C and D). Thus, while several kinds of NK1.1+ cells can be found in the livers of uninfected mice, NK cells were the main population responding to infection at day 2.

Flow cytometry was used to assess the activation status and effector function of intrahepatic lymphocytes at the protein level in *Ifnar1*^ΔHep^ mice. CD69 is an early activation marker that is induced by IFNAR signaling and is involved in the retention of lymphocytes in infected tissues (44, 45). There was a significant upregulation of CD69 expression on T and B cells from *Ifnar1*^ΔHep^ mice but not *Ifnar1*^ΔHepΔHsc^ mice (Fig. S5A), consistent with IFNAR-mediated signaling into immune cells of *Ifnar1*^ΔHep^ mice. However, few IFN-γ+ T cells were present in the livers at this time (Fig. S5B and C). In contrast, direct *ex vivo* intracellular cytokine staining (without re-stimulation) revealed elevated populations of NK cells expressing IFN-γ and granzyme B (GZMB) (Fig. 2D). Over 70% of NK1.1+ cells in *Ifnar1*^ΔHep^ mice made GZMB and ~25% produced IFN-γ, whereas few NK cells made IFN-γ or GZMB in uninfected *Ifnar1*^ΔHep^ mice or day 2-infected *Ifnar1*^ΔHepΔHsc^ mice. While the total numbers of CD45+ leukocytes and NK1.1+ cells (cells/gram of liver) were similar in *Ifnar1*^ΔHep^ and *Ifnar1*^ΔHepΔHsc^ livers (Fig. S5B), there were substantially more IFN-γ+ and GZMB+ NK cells per gram of liver in *Ifnar1*^ΔHep^ mice (Fig. 2E). Additionally, NK cell expression of NKG2D, an activating receptor induced on activated NK cells, was significantly higher for NK cells in *Ifnar1*^ΔHep^ mice compared to *Ifnar1*^ΔHepΔHsc^ mice. By comparison, CD8+ T cells expressed limited amounts of NKG2D at this time, and there was no difference between groups (Fig. S5C). These findings highlight a crucial role for IFNAR signaling in NK cells that promotes their accumulation in the infected liver and expression of antiviral effector molecules. Additionally, immunofluorescence staining with RNAscope showed co-localization of NK cells, *Ifng*, and viral RNA in *Ifnar1*^ΔHep^ liver at day 3 p.i., which was not present in an uninfected control liver section (Fig. S5D), suggesting targeted movement of activated NK cells to sites of infection. Following this initial immune activation on day 2 p.i., there was a subsequent decline in hepatic viral load and serum ALT level by day 5 p.i., suggesting that strong early NK cell responses facilitate rapid control of viruses.

### NK cell-mediated control of HAV infection

**Fig 3:**
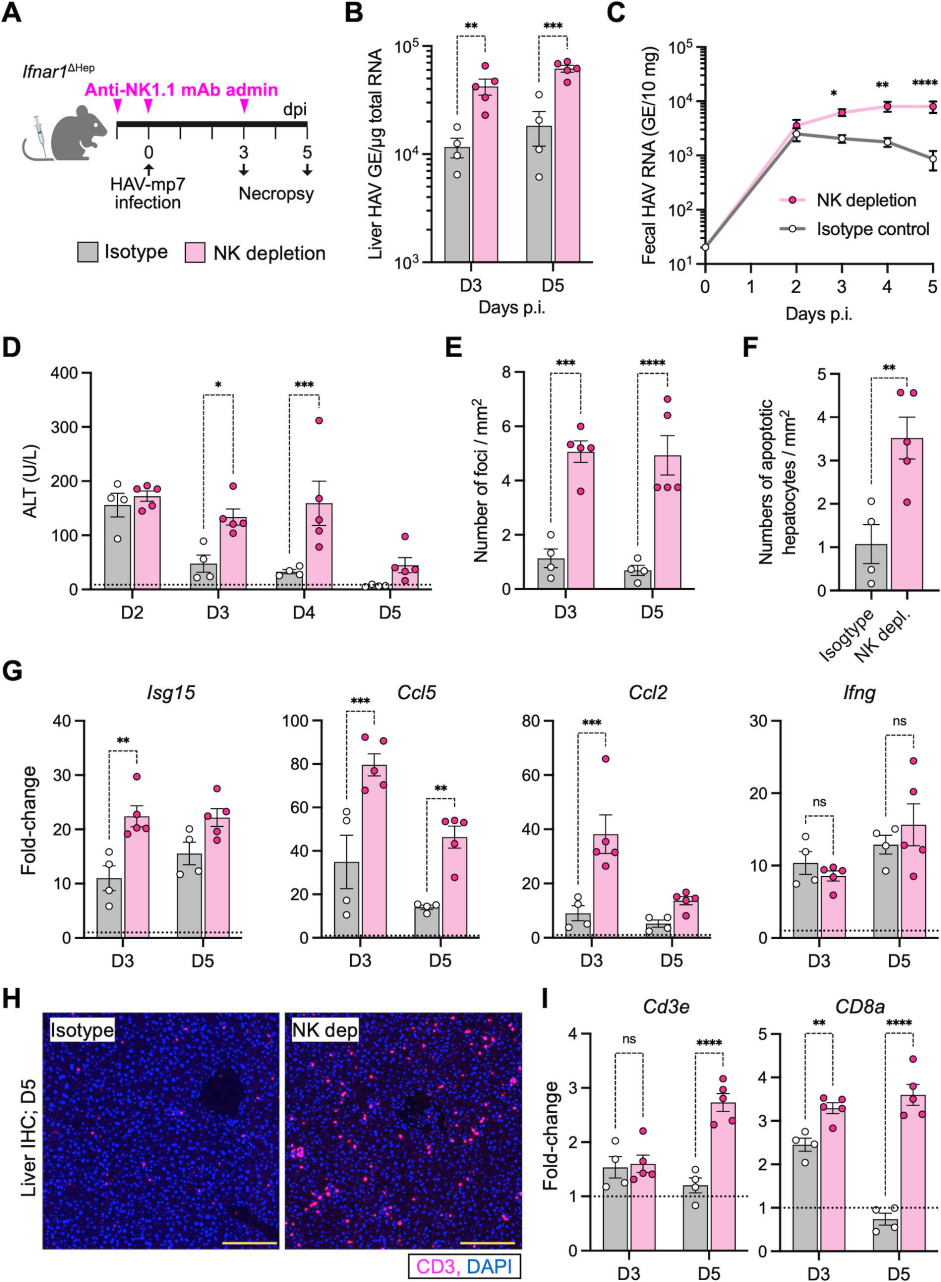
NK cell depletion increases viral RNA and serum ALT activity in *Ifnar1*^ΔHep^ mice. (**A**) *Ifnar1^ΔHep^* mice were injected intraperitoneally with 100 µg of depleting anti-NK1.1 mAb or isotype control before and after HAV infection (2 × 10^7^ GE) and necropsied at days 3 and 5 p.i. (**B**) Intrahepatic HAV RNA. (**C**) Fecal HAV shedding. (**D**) Serum ALT activity. (**E**) Liver pathology was measured according to the average number of inflammatory foci present per 1 mm^2^ of H&E-stained liver section. (**F**) Number of apoptotic hepatocytes per 1 mm^2^ in sections. (**G**) Cytokine transcript abundance in liver tissue collected at necropsy. (**H**) Immunofluorescence staining at day 5 p.i. for the T cell marker CD3. (**I**) qRT-qPCR quantification of T cell markers (*Cd3e* and *Cd8a* transcripts) in livers. The horizontal dotted lines indicate levels found in the livers of uninfected *Ifnar1^ΔHep^* mice. The data are presented as mean ± SEM, with dots indicating individual mice. Significance was assessed by two-way ANOVA with Šidák’s test for multiple comparisons (panels **B**, **C, D, E, G, and I**), or by Welch’s *t*-test for the two-group comparison in panel F. Significance is depicted as *P < 0.05; **P < 0.01; ***P < 0.001; ****P < 0.0001; and ns, *P* > 0.05.

To further investigate the functional role of IFN-responsive NK cells, we conducted *in vivo* NK cell depletion studies in HAV-infected *Ifnar1*^ΔHep^ mice. Mice were injected intraperitoneally (i.p.) with anti-NK1.1 monoclonal antibody (clone PK136) or mouse IgG2a isotype control before and after HAV infection, followed by necropsy at days 3 and 5 p.i. (Fig. 3A). NK depletion significantly increased *vRNA* in the liver and fecal HAV shedding at days 3–5 (Fig. 3B and C), which tracked with significantly higher serum ALT activity during this time (Fig. 3D). Histopathological analysis of liver sections revealed more inflammatory foci, including apoptotic hepatocytes, in NK cell-depleted mice (Fig. 3E and F; Fig. S6A), mirroring ALT activity in these mice. Finally, NK cell depletion led to increased intrahepatic expression of pro-inflammatory ISGs, including *Isg15*, *Ccl5*, *Ccl2,* and *Tnf* at day 3 and *Ccl5* at day 5 (Fig. 3G; Fig. S6B). There was no significant difference in *Ifng* mRNA levels between NK cell-depleted and control mice (Fig. 3G), perhaps due to compensation by *Ifng*-expressing NK1.1^–^ T cells or innate cell populations. The livers of NK-depleted mice had more CD3+ T cells at day 5 (Fig. 3H) and elevated expression of T cell-associated transcripts (Fig. 3I), perhaps due to the greater viral burden and expression of viral epitopes in the livers of NK-depleted mice. Unlike NK cells that co-localized to sites of infection (Fig. S5D), the CD3+ cells were not enriched at foci of inflammation and were widely distributed in the liver (Fig. 3H). In sum, these data show that NK cells control early HAV replication and limit infection-induced liver injury in *Ifnar1*^ΔHep^ mice. They contrast sharply with the results of similar studies in *Ifnar1^-/-^* mice, in which NK cell depletion had no effect on liver injury, fecal virus shedding, or intrahepatic viral RNA abundance (37). Collectively, these contrasting results show the importance of type I IFN activation of NK cells in HAV infection.

### IFN-γ limits HAV replication in hepatocyte cultures

**Fig 4:**
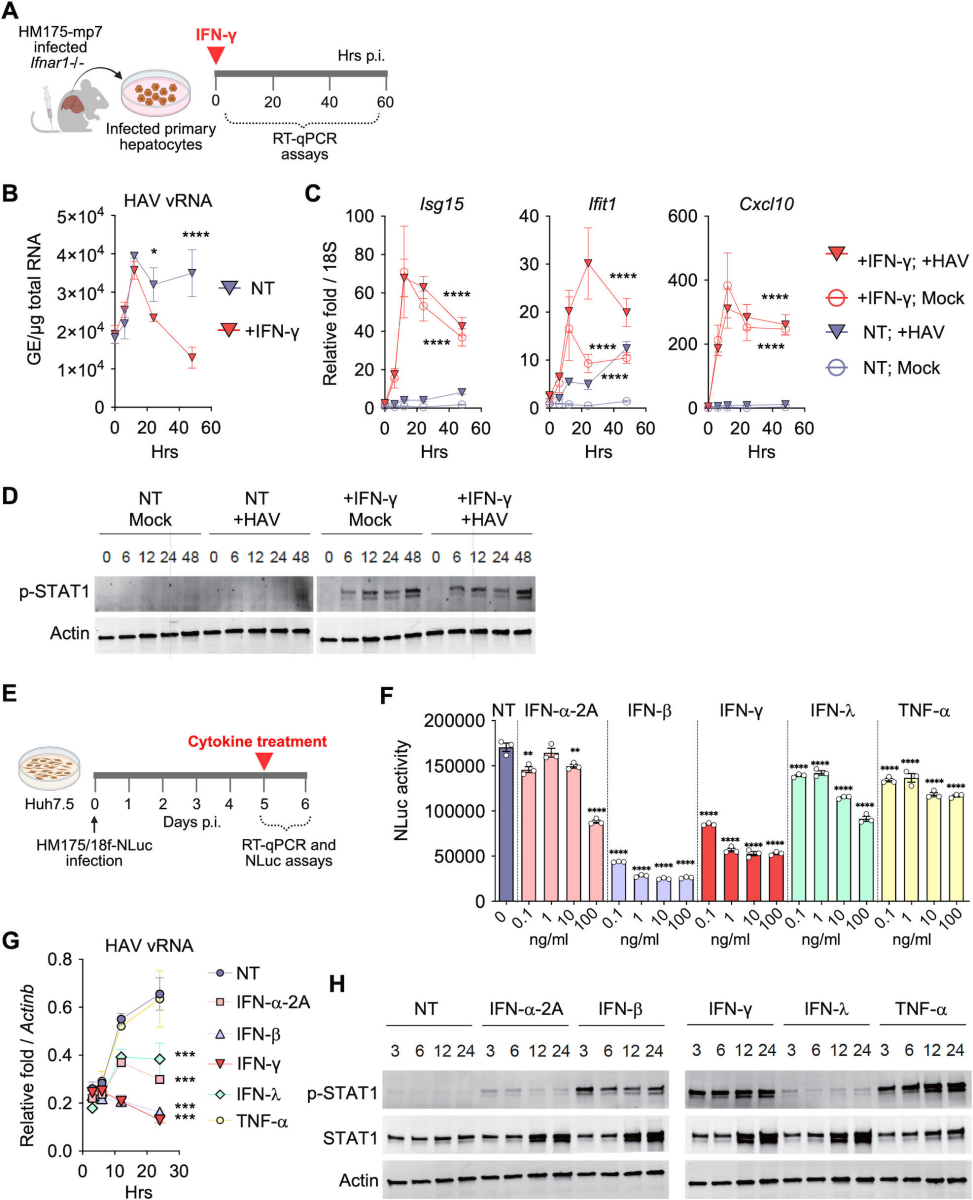
Direct antiviral effects of IFNγ against HAV *in vitro*. (**A**) Primary murine hepatocytes were isolated from 10-week-old male *Ifnar1*^-/-^ mice 24 h after i.v. challenge with HM175-mp7 (2 × 10^7^ GE). The cultured PMHs were treated with or without IFNγ (100 ng/mL) for up to 48 hours, followed by qRT-PCR quantification of viral RNA and *Isg* at multiple time points or Western analysis of actin and pSTAT1 at 48 hours. (**B**) Viral RNA levels monitored at multiple time points through the 2-day culture, normalized to *18S* mRNA. (**C**) The amount of *Isg15*, *Ifit1*, and *Cxcl10* relative to *18S* mRNA. (**D**) Immunoblot of phospho-STAT1, with actin serving as a loading control. (**E**) Huh-7.5 cells were infected with 18f-NLuc, a recombinant virus expressing a nanoluciferase reporter. At day 5, the cells were exposed to varying concentrations of several interferons for 24 hours. Virus replication was assessed by luciferase activity, *Isg* expression was quantified by qRT-PCR, and STAT1/pSTAT1 amounts were assessed by Western. (**F**) Nanoluciferase activity at 24 h following exposure to different concentrations of interferons or none added (NT, not treated). (**G**) Abundance of viral RNA relative to *β-actin* mRNA in cells treated with 100 ng/mL interferon. (**H**) Immunoblots show STAT1, pSTAT1, and actin amounts in lysates prepared from HAV-infected Huh-7.5 cells at several times post-exposure to the indicated recombinant interferon (100 ng/mL). Data are means ± s.e.m., *n* = 3 at each time point. Statistical analyses were performed using two-way ANOVA with Šídák’s test (panel** B**) or with Dunnett’s multiple comparisons test (panels** C**, **F, and G**), where interferon treatments were compared to NT or NT-mock controls. Significance is depicted as **P < 0.01; ***P < 0.001; and ****P < 0.0001.

IFNγR1 and IFNγR2 are expressed by most hematopoietic cells. Hepatocytes from WT and *Ifnar1^-/-^* mice also express *Ifngr1* and *Ifngr2* ([46] and data not shown). Based on PARSE-scRNAseq analyses, the proportion of *Ifngr* positive hepatocytes increased from ~25% in uninfected *Ifnar1^-/-^* mice to ~39% by day 5 of infection (*P* values were ~10^−22^ for *Ifngr1* and 10^−8^ for *Ifngr2*), though there was no substantial change in the average level of expression (log_2_ fold change) among *Ifngr1*+ or *Ifngr2*+ cells following infection (data not shown). We previously showed that the addition of recombinant-IFN-γ (rIFN-γ) to infected primary hepatocyte cultures from WT mice reduced the abundance of viral RNA (32). To assess the antiviral effects of IFN-γ in *Ifnar1^-/-^* cells, primary mouse hepatocytes (PMHs) were isolated from uninfected *Ifnar1*^-/-^ mice or mice pre-infected with HM175-mp7 and then exposed to rIFN-γ (Fig. 4A). Without treatment, there was an increase in viral RNA across the 48 hours of culture (Fig. 4B), which greatly exceeded what was observed for infected WT hepatocytes (32). In contrast, PMHs treated with rIFN-γ showed significantly reduced viral RNA amounts. IFN-γ increased *Isg15, Ifit1*, and *Cxcl10* expression in uninfected and virus-infected PMHs (Fig. 4C), perhaps explaining why *Ifnar1*^ΔHep^ mice made more *Isg15* and *Ifit1* than *Ifnar1*^ΔHepΔHsc^ or *Ifnar1^-/-^* mice (Fig. 1G). Interestingly, only IFN-γ-treated HAV-infected PMHs showed sustained *Ifit1* expression, suggesting that certain *Isgs* require infection-induced signals along with IFN-γ signaling to support their expression. Immunoblot analysis of PMHs confirmed that IFN-γ increased pSTAT1 levels, but there was no apparent difference between infected and uninfected PMHs in the kinetics or amount of pSTAT1 (Fig. 4D).

IFN-γ also exerted direct antiviral effects on human hepatocytes. Huh-7.5 cells, a human hepatoma-derived cell line, were infected with 18f-NLuc virus, a recombinant reporter virus that expresses nanoluciferase from a sequence inserted into the polyprotein-coding region of the genome (Fig. 4E), with luciferase activity tracking the active translation of viral RNA (47). The 18f-NLuc-infected Huh7.5 cells were then treated with different concentrations of recombinant human interferons or cytokines. Untreated cells showed increasing luciferase over 24 hours, consistent with HAV replication. However, nanoluciferase activity was significantly reduced for cells exposed to IFN-β or IFN-γ (Fig. 4F). IFN-α and IFN-λ (IL-28a) caused a weaker but dose-dependent inhibition of replication. TNF marginally reduced luciferase activity, but the effect was not clearly dose-dependent.

Viral RNA and ISG expression was quantified for Huh7.5 cells that were exposed to 100 ng/mL recombinant interferons or cytokines. There was an increase in viral RNA abundance in control-treated cells as the reporter virus replicated (Fig. 4G). However, cells treated with IFN-γ or IFN-β showed no increase in viral RNA. IFN-λ and IFN-α-2A limited the increase in viral RNA, whereas TNF had no impact on viral RNA accumulation. Immunoblot analysis at multiple time points during the 24 hour post-treatment period showed that IFN-γ and IFN-β induce STAT1 phosphorylation at Tyr701 (Fig. 4H), confirming direct activation of the JAK-STAT signaling pathway. Together, these findings demonstrate that IFN-γ directly suppresses HAV replication in both human hepatoma cells and primary mouse hepatocytes.

### IFN-γ reduces pathogenesis during HAV infection

**Fig 5:**
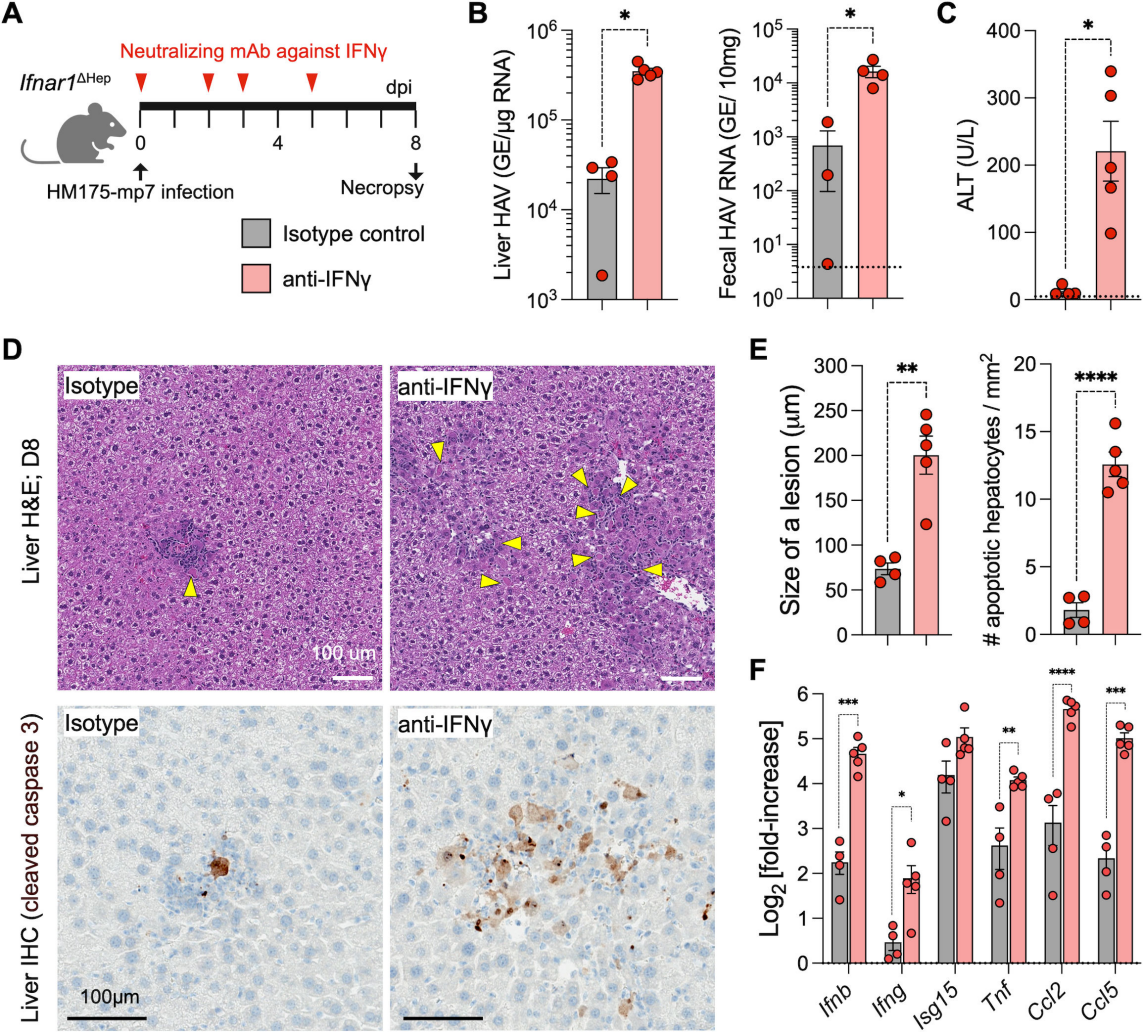
Neutralization of IFNγ increases susceptibility to HAV infection in *Ifnar1*^ΔHep^ mice. (**A**) Experimental scheme for assessing the effects of IFNγ neutralization *in vivo. Ifnar1*^ΔHep^ mice were inoculated with HM175-mp7 (2 × 10^6^ GE) and administered either 0.4 mg of anti-IFNγ mAb (*n* = 5) or isotype control mAb (*n* = 4) via i.p. injection on days 0, 2, 3, and 5. Mice were analyzed on day 8 p.i. (**B**) HAV RNA in livers (left) and in fecal pellets (right). (**C**) Serum ALT activity. (**D**) Top: representative H&E-stained liver sections (bar = 100 µµm) with yellow arrows indicating apoptotic hepatocytes; Bottom: liver sections with IHC staining for cleaved-caspase-3 (brown; black bar = 100 mm). (**E**) The bar graphs indicate (left) the average size of the inflammatory lesions in H&E-stained liver sections and (right) the number of cleaved Cas3+ apoptotic hepatocytes per mm^2^ of the scanned image view. (**F**) RT-qPCR quantification of liver transcripts normalized to *β-actin* mRNA. Horizontal dashed lines indicate the normal range for WT mice with no virus challenge. The circles in each bar graph represent data from individual animals. Significance was assessed by Mann-Whitney test (panels** B** [left] and **C**) or by *t*-test with Welch’s correction (panels B [right], E, and F), with *P < 0.05; **P < 0.01; ***P < 0.001; ****P < 0.0001.

*Ifnar1*^ΔHep^ mice showed expedited control of high-dose infection (Fig. 1), which tracked with significant increases in hepatic IFN-γ+ NK cells (Fig. 2). Removing NK cells exacerbated virus outgrowth and liver injury (Fig. 3). Because IFN-γ is made by NK cells early on (Fig. 2) and by antiviral T cells at later times (22), we sought to disrupt IFN-γ activity across an entire week of infection and examine how this impacts virus control and pathogenesis. Cohorts of infected *Ifnar1*^ΔHep^ mice were challenged with HAV and were given neutralizing anti-IFN-γ or isotype-control antibody at days 0, 2, 3, and 5 p.i., followed by analysis at day 8 (Fig. 5A). This regimen reduces IFNγR signaling throughout the period when NK cells and T cells are engaged in the immune response. The neutralization of IFN-γ significantly increased liver viral RNA and fecal shedding (Fig. 5B), indicating impaired viral control. Serum ALT levels were significantly elevated in anti-IFN-γ-treated mice (Fig. 5C), and the livers of these mice showed increases in cleaved-caspase-3 staining and more inflammatory foci (Fig. 5D and E), consistent with worsened pathology. The significant increases in liver expression of *Ifnb*, *Isg15*, *Ifng*, *Tnf*, *Ccl2*, and *Ccl5* (Fig. 5F) are consistent with enhanced virus replication in the absence of IFN-γ signaling. Collectively, these results demonstrate that IFN-γ expression in response to infection reduces replication and limits pathogenesis during HAV infection.

## DISCUSSION

NK cells serve as an initial line of defense against viral infections by recognizing infected cells and controlling virus replication through cytokine production and cytotoxic activity. NK cells in the liver are expected to play a crucial role in controlling hepatotropic viral infections due to their high numbers in this organ (48). However, the precise role of NK cells in defense against HAV has been unclear (49). Here, we characterized NK cells and IFNs in mice with an IFNAR-responsive immune system but lacking hepatocyte expression of IFNAR1. We found that these *Ifnar1*^ΔHep^ mice can support HAV replication in the liver, but the duration and magnitude of infection were greatly reduced by the actions of NK cells that responded to IFNs by rapidly expressing cytolytic molecules and IFN-γ. Additionally, we show that IFN-γ was prominently made by NK cells early on, and that it can directly limit virus replication in hepatocytes without triggering apoptosis. Our findings indicate that IFN-activated NK cells and IFN-γ ameliorate HAV-induced liver pathogenesis. Overall, this study of NK cells and our previous analysis of virus-specific T cells (22) challenge the longstanding notion that cytolytic immune cells are responsible for liver injury during HAV infection (15).

NK cells were induced to express IFN-γ and granzyme-B in HAV-infected *Ifnar1*^ΔHep^ mice but not in *Ifnar1^-/-^* or *Ifnar1*^ΔHepΔHsc^ mice. This also tracked with their increased abundance in the liver early after infection, indicating that direct type-1 IFN signaling in NK cells is essential for their rapid reaction to HAV. Our data show that infection and pathogenesis increase when IFN-responsive NK cells are experimentally depleted. In contrast, NK cell depletion in *Ifnar1^-/-^* mice failed to increase infection (37), most likely because their NK cells could not be activated by IFNAR signaling. NK cells show rapid antiviral effects at these early times; however, there are distinct subsets of NK cells, and our analyses did not distinguish immune responses due to liver-resident cells versus infiltrating populations. There may be a role for NK cells in homeostasis or liver repair, as inferred from NK cell studies in HCV-infected individuals (50). As noted previously, there is considerable heterogeneity in NK cell subsets within an individual and between individuals due to the large variety of activating and inhibiting NK receptors and allelic diversity across populations (31). A comparison of NK receptors and NK subsets in subjects who resolve acute HAV versus those who develop fulminant hepatitis may provide insights into how NK cells may impact these divergent outcomes.

We noted an increase in the frequency of T cells in the livers of infected NK-depleted *Ifnar1*^ΔHep^ mice at day 5 compared to NK-replete mice, based on immunofluorescence staining for CD3 and RT-qPCR quantification of *Cd3* and *Cd8a*. It may be that without NK cells, there is increased virus replication and associated expression of viral epitopes and chemokines, leading to more T cell recruitment or local proliferation of T cells in the liver. However, this outcome could also be explained by NK cells killing or otherwise impairing the functions of APCs or effector T cells in this model, as described in other virus infections (28, 29, 51–60). This observation illustrates how interpretations of virological and clinical data can be confounded by limited analyses of immune cell populations in the blood. An enhanced NK response in blood could represent vigorous innate defenses, but it may also track with weakened adaptive immunity and consequent viral pathogenesis.

NK cells were effectively depleted by anti-NK1.1 antibody (PK136) (Fig. S6B), but the treatment may have also eliminated other NK1.1+ cells, including ILC1s, NKT cells, and subsets of activated T cells that are induced to express NK1.1 (61–69). While we have not formally ruled out antiviral roles for these other cell types, analyses of scRNAseq data (Fig. S4) did not reveal substantial populations of NK1.1^+^ ILC1 cells (*Klrb1^+^Itga1^+^Tbx21^+^Eomes^–^*) or NKT1 cells (*Klrb1^+^Cd3^+^Plzf^+^Tbx21^+^*) that made *Ifng* or *Gzmb* at day 2 post-infection. While these cells might be involved in reactions to infection within the first 2 days, the effect of NK cell depletion on viral burdens was more apparent after day 2 when the frequency of ILC1 and NKT1 cells was low. Thus, we infer that the effects of the PK136 antibody on viral burdens, ALT, and inflammatory foci were due to the absence of protection by NK cells. The transcriptomic data revealed a few T cells that expressed NK1.1 (*Klrb1^+^Cd3^+^Plzf^–^*) and made these effector molecule transcripts; however, their intensity of effector gene expression was lower than that of authentic NK cells, implying that activated NK cells are more important for immune control at these early times.

Bulk RT-PCR (Fig. 3G; Fig. S6B) identified *Ifng* and *Tnf* in NK-depleted livers, suggesting that residual NK1.1-negative subsets of activated T cells, non-classical NKT cells, or MAIT cells could be sources of these transcripts. Interestingly, while CD8+ T cells were increased in the absence of NK1.1+ cells at days 3–5 (Fig. 3H and I) and likely were a source of these cytokines, these cells failed to localize to foci where infected and apoptotic cells are found and were instead spread diffusely in the liver. There was an increase in viral RNA in NK-depleted mice (Fig. 3B and C), indicating that the elevated population of CD8+ T cells in NK-depleted mice was incapable of restricting infection. This contrasts with NK-replete mice, where NK cells and *Ifng* were found in close juxtaposition to infected cells (Fig. S5D), suggesting that virus control depends on targeted delivery and locally high concentrations of effector molecules near cells replicating virus. It is noteworthy that the antiviral effects of NK cells were associated with declining levels of ALT, not immune-driven pathogenesis.

One caveat with our study is that only male mice were characterized. Male mice generate substantially stronger inflammatory responses and reach higher ALT levels than age-matched female mice infected with HAV (22). This suggests that male mice may have weaker immune responses, reduced IFN-γ production, and higher viral loads compared to females. Epidemiologically, the incidence of severe disease is greater in men than women (70–74). The impact of sex differences on antiviral NK cell responses has been understudied. pDCs from women may produce more type I IFN, and female NK cells can show greater responsiveness to IFN (75). Such sex-based differences in immunity can arise from multiple mechanisms. First, sex hormones can act directly on immune cells and impact their reactivity or alter cell-intrinsic antiviral defenses (76). In addition to hormonal influences, cell-intrinsic genetic and epigenetic differences also contribute to sex-biased immune responses. Several immune-related genes are located on the X chromosome and may escape X inactivation, resulting in higher expression in females. One such gene is *Kdm6a* (aka, UTX), which is a histone demethylase and key epigenetic regulator involved in increasing the accessibility of enhancers. Cheng et al. (77) demonstrated that while the number of NK cells increases in male mice, their effector functions are reduced compared to females, which is a characteristic also found in humans. These functional differences were attributed in part to lower UTX expression in male NK cells, which contributed to increased susceptibility to MCMV infection in male mice. In future studies, it would be important to investigate whether there are sex-dependent differences in early NK cell responses that impact the set point of HAV infection.

Another caveat with our study is that the temporal features of infection and consequent immune response are accelerated compared to those typically observed in humans or primate models (1, 5, 78), where there is an extended 3–4 week delay following initial infection before symptoms of liver injury emerge. Throughout this study, we administered HAV intravenously at a high dose, and it may be that giving lower amounts would delay the peak of infection. We surmise that patients are typically exposed to relatively limited amounts of virus orally, rather than high doses intravenously, resulting in the characteristic 3-week prodromic phase. It would be informative in future studies to assess side-by-side how different infection doses, routes, and infection kinetics impact immune cell responses to infection.

We found that IFN-γ plays a critical role in directly controlling HAV replication and mitigating liver damage. Neutralizing IFN-γ significantly increased liver viral RNA and fecal virus shedding, along with elevated serum ALT levels in *Ifnar1*^ΔHep^ mice. IFN-γ induced *Isgs* in human hepatoma and mouse primary hepatocytes. IFN-γ was expressed at higher levels in *Ifnar1*^ΔHep^ mice compared to *Ifnar1^-/-^* or *Ifnar1*^ΔHepΔHsc^ mice, which may explain the elevated ISG response in these mice that restricted infection. IFN-γ did not directly induce apoptosis. Rather, the neutralization of IFN-γ increased viral replication, which in turn resulted in a higher number of apoptotic cells *in vivo*. Our previous studies using *Ifnar1^-/-^* mice demonstrated that unconstrained virus replication triggers MAVS:pIRF3-dependent transcriptional responses that lead to cell death (32, 37, 39), and our findings here suggest that IFN-γ supports ISG responses that limit virus replication without triggering hepatocyte apoptosis. IFN-γ robustly induces the expression of IRF1, a key component of constitutive antiviral defenses against HAV at early stages of infection (47, 79). The precise mechanisms by which IFN-γ signaling restricts replication, or supports immune cell activity, remain incompletely understood. These findings highlight the critical role of IFN-γ in the early stages of HAV infection and may contribute to the development of novel therapeutic strategies for ameliorating liver injury in severe HAV infections.

## MATERIALS AND METHODS

### Mice

*Ifnar1^-/-^* mice (80) were backcrossed to B6-derivative mice at TSRI (La Jolla, CA, USA) and at UNC-Chapel Hill. *Albumin*-Cre mice (JAX#003574), *Ifnar1*^fl/fl^ mice (JAX#028256), and *Vav^Cre^* (JAX#008610) mice were purchased from JAX and bred in-house at UNC. *Ifnar1*^ΔHep^ mice that lack *Ifnar1* specifically on hepatocytes were generated through intercrosses of *Albumin*-Cre mice and *Ifnar1*^fl/fl^ mice. *Ifnar1*^ΔHepΔHsc^ mice, which are genetically deficient for *Ifnar1* in hepatocytes and hematopoietic stem cells, were generated through intercrosses of *Albumin*^Cre^, *Vav^Cre^*, and *Ifnar1*^fl/fl^ mice. For HAV infections, cohorts of 8–12-week-old mice were given 2 × 10^6^ or 2 × 10^7^ genome equivalents of mouse-passaged HAV by intravenous injection. Fecal pellets were collected from mice housed in individual cages. Serum samples were collected at multiple time points for quantification of blood ALT activity. Tissues were harvested at necropsy and stored in RNAlater (ThermoFisher Scientific), snap frozen on dry ice, and kept at −80°C, or fixed in 10% neutral phosphate-buffered formalin for 48 h and subsequently stored in 70% ethanol until processed for histology. Male and female bone marrow chimera mice were generated by lethally irradiating recipient mice (CD45^.2/.2^) twice at 600 Rads across a 4-hour period, followed by the intravenous transfer of 1.2 × 10^7^ bone marrow cells from donor mice (CD45^.1/.2^). The recipient mice were given drinking water containing sulfamethoxazole (6 mg/mL) for 2 weeks and allowed to establish immune homeostasis across 8 weeks. The efficiency of reconstitution was assessed by surface staining of blood leukocytes with antibodies against CD45.1 and CD45.2. Mice that were successfully reconstituted were challenged with HAV as described.

### Virus

Mice were challenged with mp7 HM175, a wild-type strain (Human/Australia/HM175/1976) of HAV that was serially passaged first in *Ifnar1*^-/-^*Ifngr1*^-/-^ mice and then in *Mavs*^-/-^ mice for a total of seven mouse passages (37). The inocula were prepared from the liver homogenates of HAV-infected *Mavs^-/-^* mice, as described (37). HM175/18f-NLuc was generated by cloning the nano-luciferase gene into the HAV-VP1:pX gene in an infectious molecular clone of HM175/18f virus (47). Huh7.5 cells were infected with 10 MOI (GE/cell) of HM175/18f-NLuc, as described previously (47).

### Infection of hepatoma cells

Huh7.5 cells are immortalized human hepatoma cells. These cells are cultured in high-glucose DMEM, supplemented with 10% FBS, GlutaMAX-1, penicillin and streptomycin, and non-essential amino acids (Thermo Fisher Scientific). For infections, Huh7.5 cells were split, cultured 1 day in 10% DMEM media, and then infected with 10 MOI of HM175/18f-NLuc, as described previously (47). Unbound virus was washed away after 1 hour of adsorption, and fresh media were added. At day 5 post-infection, interferons were added to some cultures. Cell lysates were collected at several times within a 24-hour period for qRT-PCR analyses of viral RNA or *Isg* and the housekeeping gene *β-actin* transcripts. NanoLuc activity was assessed at 24 hours using a Luciferase assay.

### Luciferase assay

Cells were lysed in a 1× passive lysis buffer (Promega, #E1941) for 15 min at room temperature, and lysates were transferred to opaque white 96-well plates (Corning, #3912). NLuc assays were carried out with the NLuc GLOW Assay kit (Nanolight Technology, #325), and FLuc and dual luciferase assays were carried out with the Dual Luciferase Assay Kit (Promega, #E1910). Luminescence was measured using a BioTek Synergy II multimode plate reader (BioTek Instruments).

### Alanine transferase

Mouse sera (2.5 µL) were diluted 1:2 in PBS and assayed for ALT activity using the Alanine Aminotransferase Activity Assay kit (Elabscience, #E-BC-K235-M).

### Histology

Tissues were fixed in 10% neutral phosphate-buffered formalin for 48 hours and stored in 70% ethanol until processed for histology at the Pathology Services Core Facility at UNC-CH. Formalin-fixed paraffin-embedded tissues were sectioned at 5 µm thickness and stained with hematoxylin and eosin (H&E), or were stained for immunofluorescence microscopy using anti-CD3 (Cell Signaling Technologies, #85061s) with Hoechst-33258 (Invitrogen, #H3569), or using anti-cleaved caspase-3 antibody (Cell Signaling, #9664) with Mayers Hematoxylin counterstain. Some sections were stained for immunofluorescence using anti-CD68 (Abcam, #125212) or anti-NK1.1 (Cell Signaling, #39197), with fluorescent RNAscope 2.5 LS probes for HAV-RNA (ACDBio, #1115888C) or *Ifng* mRNA (ACDBio, #1045168C) according to the manufacturer’s protocol, along with DAPI. H&E- and cleaved-caspase-3-stained slides were scanned at 20× magnification using the ScanScope AT2 system (Leica Biosystems) at the UNC Pathology Services Core Facility. Immunofluorescence-stained slides were scanned at the same magnification using the ScanScope FL system (Leica Biosystems). All scanned images were uploaded to the eSlide Manager platform (Leica Biosystems) for analysis. Liver injury was assessed by quantifying the number of inflammatory foci and their size within 1 mm^2^ regions using eSlide Manager.

### Antibody treatments of mice

NK cells were depleted in mice by the i.p. delivery of 100 µg anti-NK1.1 (clone PK136, BioXcell) at days −1, 0, and +3 of infection. Control mice were given 100 µg non-depleting, isotype control antibody IgG2a (C1.18.4, BioXcell). IFNγ activity was disrupted by administering 0.4 mg of neutralizing anti-IFN-γ mAb (clone XMG1.2, BioXCell) via i.p. injection at days 0, 2, 3, and 5 p.i. Control-treated mice received isotype-matched antibody (clone HRPN, BioXcell) following the same schedule.

### Primary mouse hepatocytes

Hepatocytes were isolated from 10-week-old male mice 10 hours after intravenous HAV challenge. As previously described (81), mice were euthanized, the inferior vena cava was cannulated, and the liver was sequentially perfused with 0.5 M EDTA followed by collagenase solution (Liberase, Sigma-Aldrich) using a peristaltic pump. The liver was then excised, and hepatocytes were purified by Percoll-based density gradient centrifugation. Viable hepatocytes (~95%, 2 × 10⁵ cells/well) were plated onto collagen-coated 12-well plates (Corning BioCoat Collagen). Within 24 hours, most cells exhibited the characteristic hexagonal “chicken-wire” morphology of hepatocytes.

### Liver leukocytes

Livers were perfused with 10 mL of PBS, cut into small pieces, and digested at 37°C for 30 minutes with Collagenase IV (Gibco/Thermo Scientific) at 100 U/mL and 10 mg/mL DNase-I (Sigma-Aldrich) in DMEM containing 1 mM CaCl_2_ and 1 mM MgCl_2_. The liver cells were disrupted through a 70 µm strainer and then briefly centrifuged at 50 *× g* for 2 minutes to pellet residual hepatocytes, which were discarded. The clarified cell suspension was diluted into 45% isotonic Percoll solution (GE Healthcare), underlaid with a cushion of 80% Percoll, and centrifuged at 800 *× g* for 20 minutes at room temperature. The leukocytes were isolated from the interphase and were washed and resuspended in 10% RPMI.

### Blood leukocytes

Blood was isolated using heparinized capillary tubes and collected into 4% sodium citrate. The blood cells were underlaid with Ficoll, centrifuged for 20 minutes at 1,200 *× g*, and leukocytes were isolated from the interphase. The cells were rinsed and resuspended in 10% RPMI media.

### Flow cytometry

Single cell suspensions of liver were surface stained directly *ex vivo* with fluorochrome-conjugated antibodies in the presence of unlabeled antibodies against Fc-receptors. For intracellular staining, cells were fixed with fixation buffer (BioLegend; #420801) and permeabilized using Permeabilization Wash buffer (BioLegend; #421002) and incubated with antibodies against IFN-γ and Granzyme-B. NK cells and T cells were analyzed directly *ex vivo* with no restimulation. Antibody-bound cells were quantified using LSR-II or LSR-Fortessa (BD Biosciences) cytometers, and the resulting data were analyzed using FlowJo software. Hierarchical gates were established by first assessing forward- and side-scatter size, discriminating live cells using a viability dye, resolving singlets by comparing forward-scatter height and width, and then identifying specific cell populations using negative-control isotype-stained cells, FMOs, and biological controls.

### Cell preparation for single-cell RNA-seq

Intrahepatic CD45^+^ leukocytes and hepatocytes were isolated from the livers of two uninfected and two infected *Ifnar1*^ΔHep^ mice (day 2 p.i.) following a previously described protocol (81). Mice were euthanized using the isoflurane drop method. The inferior vena cava was cannulated, and the liver was sequentially perfused with 0.5 M EDTA and collagenase solutions (Liberase, Sigma-Aldrich) using a peristaltic pump. The liver was then harvested, and viable hepatocytes and leukocytes were separated by density-based centrifugation with Percoll. Cells were filtered through a 70 µm strainer and collected into separate tubes for staining and sorting. Leukocytes were stained with APC anti-mouse CD45.2 (BioLegend). Dead cells were identified using SYTOX Blue Dead Cell Stain (ThermoFisher, #S34857) and Annexin-V (BioLegend, #640918). Each sample was tagged with TotalSeq-B anti-mouse hashtag antibodies 1–4 (BioLegend, #155831, #155833, #155835, and #155837) to allow individual mouse identification. Leukocytes were sorted as CD45^+^ live cells, while hepatocytes were sorted as live cells via a Sony SH800Z cell sorter. The final yield was approximately 4,000 CD45^+^ leukocytes and 1,000 hepatocytes per mouse. Post-sort single-cell viability, assessed using propidium iodide/acridine orange staining on a Luna-FX7 cell counter, was around 85% for leukocytes and 56% for hepatocytes. All eight sorted samples (four leukocyte and four hepatocyte samples) were pooled into a single cell suspension, maintaining an 80:20 ratio of immune cells to hepatocytes. The pooled sample was counted and prepared for single-cell capture, reverse transcription, and library preparation using the Chromium Next GEM Single Cell 3′ version 3.1 kit at the UNC-CGIBD Advanced Analytics Core. This pooled suspension was loaded into two separate 10× Genomics inlets to generate parallel data sets. This multiplexed library was then loaded into a NextSeq 2000 P3 flow cell (Illumina) for paired-end sequencing at the UNC High-Throughput Sequencing Facility.

### Single-cell RNAseq data analysis

Reads were mapped to the genome build GRcM38 and demultiplexed through the 10× Genomics Cell Ranger pipeline (version 8.0.0). Raw unique molecular identifier (UMI) counts for each demultiplexed sample were imported into R (version 4.4.2) for analysis using Seurat (version 5.1.0). We excluded cells with gene counts less than 200 or more than 7,000 and kept cells with mitochondrial RNA reads < 10%. Raw UMI counts were log normalized using the LogNormalize method. Dimensionality reduction by t-distributed stochastic neighbor embedding (tSNE) was performed following PCA reduction using 10 principal components. The data were clustered with the Louvain with multilevel refinement algorithm in Seurat at a resolution of 0.5. Clusters were visualized by tSNE plots. DESeq2 was used to compare differential expression between uninfected and day 2 post-infected cells. Genes with a false discovery rate-adjusted *P*-value less than 0.05 were considered significantly differentially expressed. Cell communication analysis was conducted using the CellChat package (version 2.1.0) (82).

### Quantification of HAV RNA in liver and fecal samples

Total RNA from fecal samples was extracted using the QiaAmp Viral RNA Isolation Kit (Qiagen, Valencia, CA, USA). Total RNA from liver samples was isolated using TRIzol Reagent (Invitrogen Life Technologies, Carlsbad, CA, USA), followed by RNA purification using the RNeasy Mini Kit (Qiagen). A NanoDrop (Thermo Scientific, Wilmington, DE, USA) was used to measure RNA concentration. HAV genomic RNA was quantified by a two-step reverse-transcription quantitative PCR procedure using Super-Script-III First-Strand Synthesis Supermix (Invitrogen) and iTaq SYBR Green Supermix (Bio-Rad) and a CFX96 Real-Time PCR Detection System (Bio-Rad). HAV-specific primer sequences were 5′ GGTAGGCTACGGGTGAAAC-3′and 5′-AACAACTCACCAATATCCGC-3′ (37). HAV RNA levels were determined by reference to a standard curve generated with synthetic HAV RNA.

### qRT-PCR

Viral RNA, chemokine, and interferon-stimulated gene expression were quantified using qRT-PCR, as described previously (37).

### Immunoblots

Lysates from infected Huh7.5 cells were separated via 4%–15% SDS-PAGE, followed by transfer to PVDF membrane and immunoblotted using antibodies against STAT1 (Cell Signaling Technology, #9172), pSTAT1 at Tyr701 (clone 58D6, Cell Signaling Technology, #9167), or actin. The bands were visualized on an Odyssey CLx Infrared Imaging System (LI-COR Biosciences).

### Statistics

The Shapiro–Wilk test was used to assess data normality. For two‐group comparisons, statistical significance was determined using unpaired, two‐tailed tests; normally distributed data were analyzed by *t*-test with Welch’s correction, and non-normal data were assessed by Mann–Whitney *U*-test. For ≥3 groups, one-way or two-way analysis of variance was performed, followed by either Šídák’s multiple comparisons test or Dunnett’s test as indicated in the figure legends. Viral titers were log_10_-transformed prior to assessing normality and performing the statistical tests. Calculations were performed using GraphPad Prism 10.5. Significance thresholds are depicted in figure legends as *P < 0.05; **P < 0.01; ***P < 0.001; ****P < 0.0001; and not significant: *P* > 0.05. All values are presented as mean ± SEM with individual data points shown.

## Data Availability

The scRNA-seq data presented in Fig. 2 and Fig. S3 and S4 have been deposited in the NCBI Gene Expression Omnibus repository with accession number GSE296163.

## SUPPLEMENTAL MATERIAL

Figure S1 jvi.01395-25-s0001.pdf Chimeric *Ifnar1*^-/-^ mice with WT (*Ifnar1*^+/+^) hematopoietic cells reduce liver injury and viral loads in HAV infection.

Figure S2 jvi.01395-25-s0002.pdf HAV infection of cKO mice lacking IFNAR1 on hepatocytes.

Figure S3 jvi.01395-25-s0003.pdf Immune cells may communicate by secreting cytokines and chemokines during the early stages of HAV infection.

Figure S4 jvi.01395-25-s0004.pdf NK1.1+ subsets in the livers of *Ifnar1*^ΔHep^ mice before and 2 days after HAV infection.

Figure S5 jvi.01395-25-s0005.pdf Immune responses in the livers of *Ifnar1*^ΔHep^ mice during HAV infection.

Figure S6 jvi.01395-25-s0006.pdf NK cells limit liver injury during HAV infection.

ASM does not own the copyrights to Supplemental Material that may be linked to, or accessed through, an article. The authors have granted ASM a non-exclusive, world-wide license to publish the Supplemental Material files. Please contact the corresponding author directly for reuse.
