# Supplementary material for: Natural killer cells and IFN-γ protect against liver injury during HAV infection in mice
Source: J Virol. 2025 Sep 19;99(10):e01395-25. doi: 10.1128/jvi.01395-25 (PMC12548451; doi:10.1128/jvi.01395-25)
Supplement: Figure S1 — Chimeric Ifnar1-/- mice with WT (Ifnar1+/+) hematopoietic cells reduce liver injury and viral loads in HAV infection. [file jvi.01395-25-s0001.pdf]

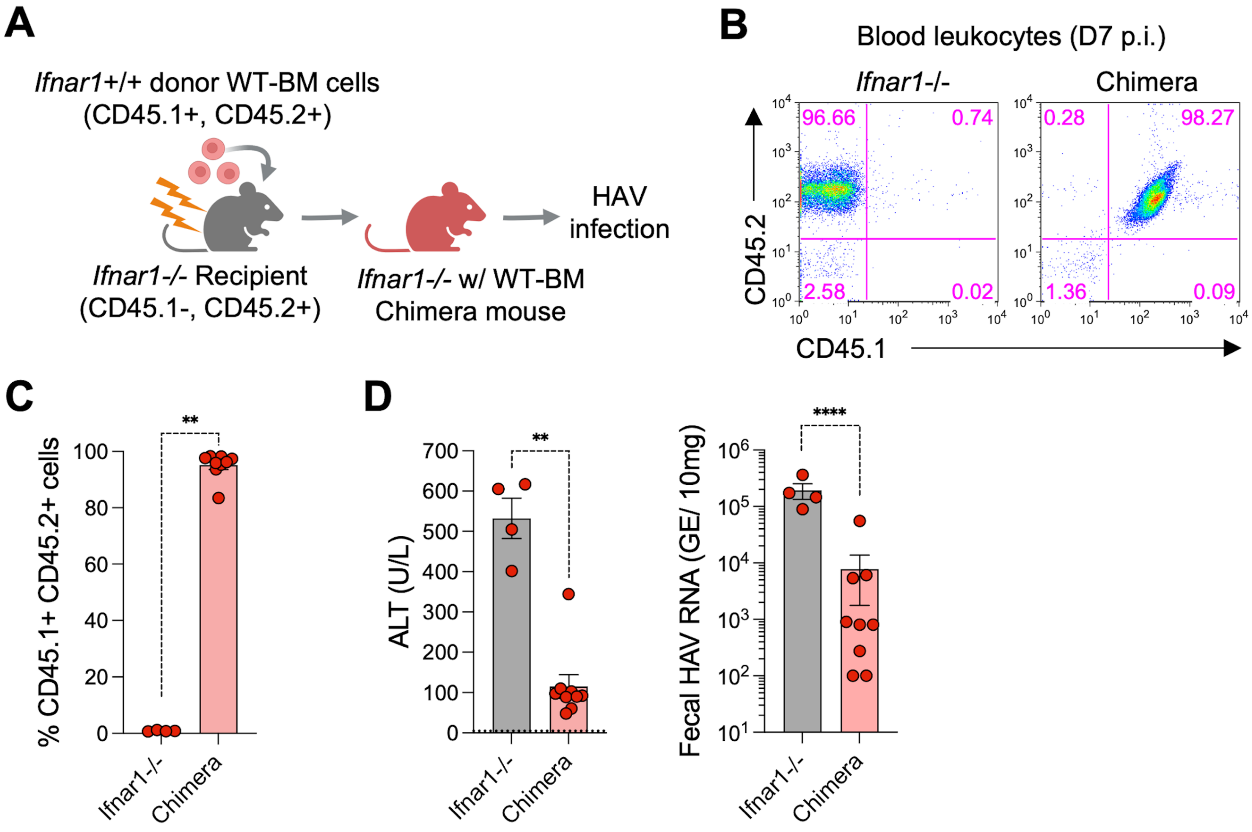

Figure S1. Chimeric *Ifnar1*-/- mice with WT (*Ifnar1*+/+) hematopoietic cells reduce liver injury and viral loads in HAV infection. (A) Recipient *Ifnar1*-/- mice (CD45.1-/-) were irradiated and reconstituted with the bone marrow (BM) cells isolated from *Ifnar1*+/+ B6.WT mice (donors; CD45.1+/-). Chimera mice (n=9) and non-irradiated *Ifnar1*-/- control mice (n=4) were infected with HM175-mp6 ($2x10^6$ GE). **(B,C)** At day 7 p.i. (60 days post-irradiation), blood from the chimera mice showed chimerism, with ~95% of CD45+ leukocytes derived from donor bone marrow. Representative FACS plots and a bar graph show the proportion of CD45.1+CD45.2+ donor cells among all blood leukocytes. (**D**) Serum ALT activity and fecal HAV shedding at day 7 p.i. Significance was assessed by Mann-Whitney test (panel C and panel D, left), or by *t*-test with Welch's correction (panel D, right). Symbols indicate individual animals.
