# Supplementary material for: Natural killer cells and IFN-γ protect against liver injury during HAV infection in mice
Source: J Virol. 2025 Sep 19;99(10):e01395-25. doi: 10.1128/jvi.01395-25 (PMC12548451; doi:10.1128/jvi.01395-25)
Supplement: Figure S2 — HAV infection of cKO mice lacking IFNAR1 on hepatocytes. [file jvi.01395-25-s0002.pdf]

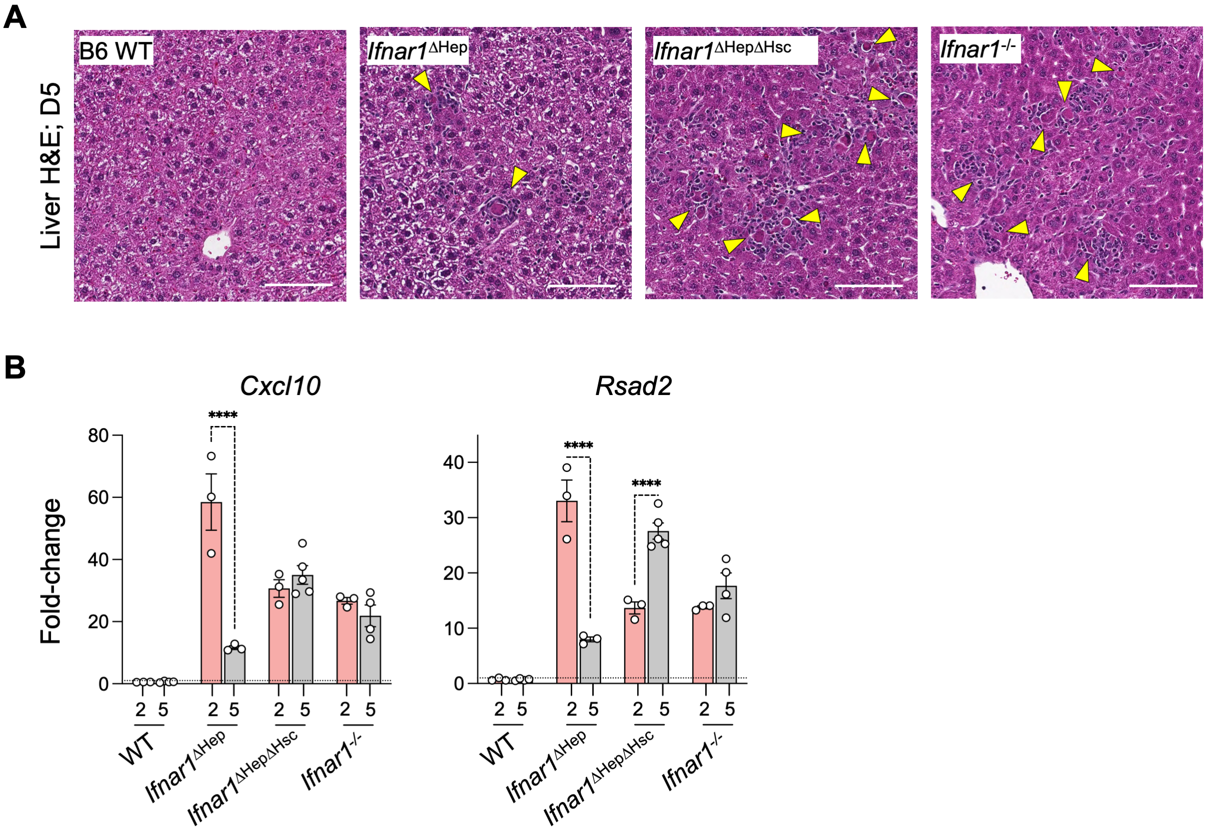

Figure S2. HAV infection of cKO mice lacking IFNAR1 on hepatocytes. WT, *Ifnar1*$^{\triangle Hep}$, *Ifnar1*$^{\triangle Hep \triangle Hsc}$, and *Ifnar1-/-* mice were challenged i.v. with $2x10^7$ GE of HM175-mp7. **(A)** Representative H&E-stained liver sections at day 5 p.i. Yellow arrows indicate inflammatory foci and apoptotic hepatocytes. **(B)** qRT-PCR quantification of *Cxcl10* and *Rsad2* transcripts in livers. Transcript abundance was normalized to *β-actin* mRNA in the same samples. The bar graphs show means ± SEM, with each dot representing an individual mouse. The horizontal dashed lines indicate levels found in uninfected, naive WT livers. Statistical testing for panel B was done by 2-way ANOVA with Šídák's multiple comparisons test.
