# Supplementary material for: Natural killer cells and IFN-γ protect against liver injury during HAV infection in mice
Source: J Virol. 2025 Sep 19;99(10):e01395-25. doi: 10.1128/jvi.01395-25 (PMC12548451; doi:10.1128/jvi.01395-25)
Supplement: Figure S3 — Immune cells may communicate by secreting cytokines and chemokines during the early stages of HAV infection. [file jvi.01395-25-s0003.pdf]

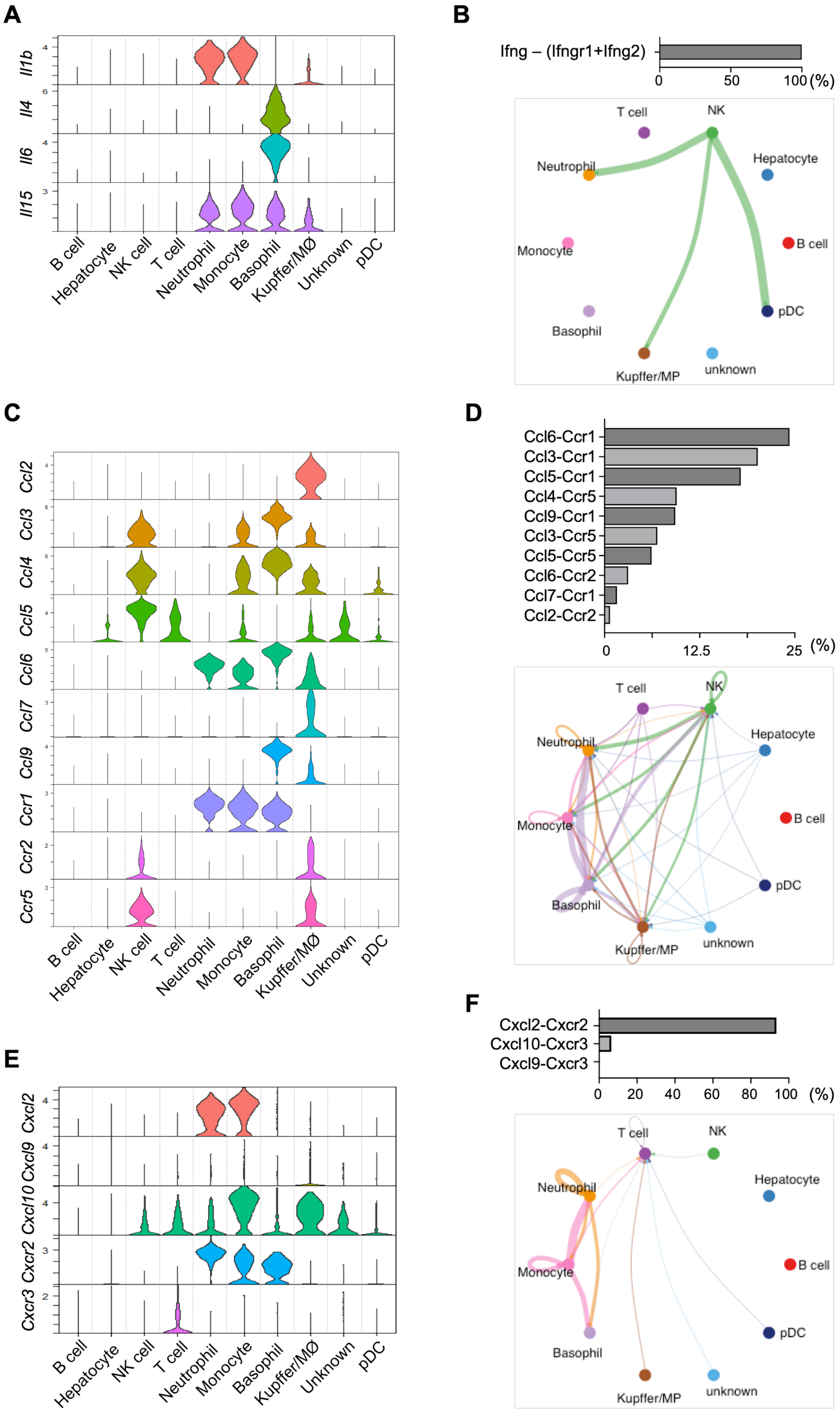
A
Il1b
Il4
Il6
Il15
B cell
Hepatocyte
NK cell
T cell
Neutrophil
Monocyte
Basophil
Kupffer/MØ
Unknown
pDC
B
Ifng – (Ifngr1+Ifng2)
0
50
100 (%)
T cell
NK
Neutrophil
Hepatocyte
Monocyte
B cell
Basophil
pDC
Kupffer/MP
unknown
C
Ccl2
Ccl3
Ccl4
Ccl5
Ccl6
Ccl7
Ccl9
Ccr1
Ccr2
Ccr5
B cell
Hepatocyte
NK cell
T cell
Neutrophil
Monocyte
Basophil
Kupffer/MØ
Unknown
pDC
D
Ccl6-Ccr1
Ccl3-Ccr1
Ccl5-Ccr1
Ccl4-Ccr5
Ccl9-Ccr1
Ccl3-Ccr5
Ccl5-Ccr5
Ccl6-Ccr2
Ccl7-Ccr1
Ccl2-Ccr2
0
12.5
25 (%)
T cell
NK
Neutrophil
Hepatocyte
Monocyte
B cell
Basophil
pDC
Kupffer/MP
unknown
E
Cxcl2
Cxcl9
Cxcl10
Cxcr2
Cxcr3
B cell
Hepatocyte
NK cell
T cell
Neutrophil
Monocyte
Basophil
Kupffer/MØ
Unknown
pDC
F
Cxcl2-Cxcr2
Cxcl10-Cxcr3
Cxcl9-Cxcr3
0
20
40
60
80
100 (%)
T cell
NK
Neutrophil
Hepatocyte
Monocyte
B cell
Basophil
pDC
Kupffer/MP
unknown

Figure S3. Immune cells may communicate by secreting cytokines and chemokines during the early stages of HAV infection. The cell populations identified by scRNAseq in HAV-infected *Ifnar1*$^{\Delta Hep}$ livers at day 2 post-infection were analyzed for their expression of cytokines, chemokines, and associated receptors. **(A)** Violin plots show the expression levels of key cytokine genes in liver cells identified by t-SNE plot (Fig. 2A). **(B)** A bar graph depicts the relative contribution of IFNγ and its receptor on the overall communication network of the IFNγ signaling pathway. A CellChat analysis of these transcripts (bottom) predicts an intercellular communication network based on IFNγ:IFNγR interactions. The line thickness represents the strength of signaling. **(C)** Violin plots show expression levels of CCL- and CCR- associated genes among liver cells. **(D)** The bar graphs show the relative contribution of each indicated L-R pair on the overall CCL signaling network. The bottom graphic shows a CellChat-predicted intercellular communication network for CCL signaling among liver cells. **(E)** Violin plots show expression levels of CXCL and CXCR associated genes among liver cells. **(F)** The bar graph shows the relative contribution of each L-R pair for the overall CXCL signaling pathway. The bottom image depicts the CellChat-predicted intercellular communication network based on CXCL signaling interactions.
