# Supplementary material for: Natural killer cells and IFN-γ protect against liver injury during HAV infection in mice
Source: J Virol. 2025 Sep 19;99(10):e01395-25. doi: 10.1128/jvi.01395-25 (PMC12548451; doi:10.1128/jvi.01395-25)
Supplement: Figure S4 — NK1.1+ subsets in the livers of Ifnar1ΔHep mice before and 2 days after HAV infection. [file jvi.01395-25-s0004.pdf]

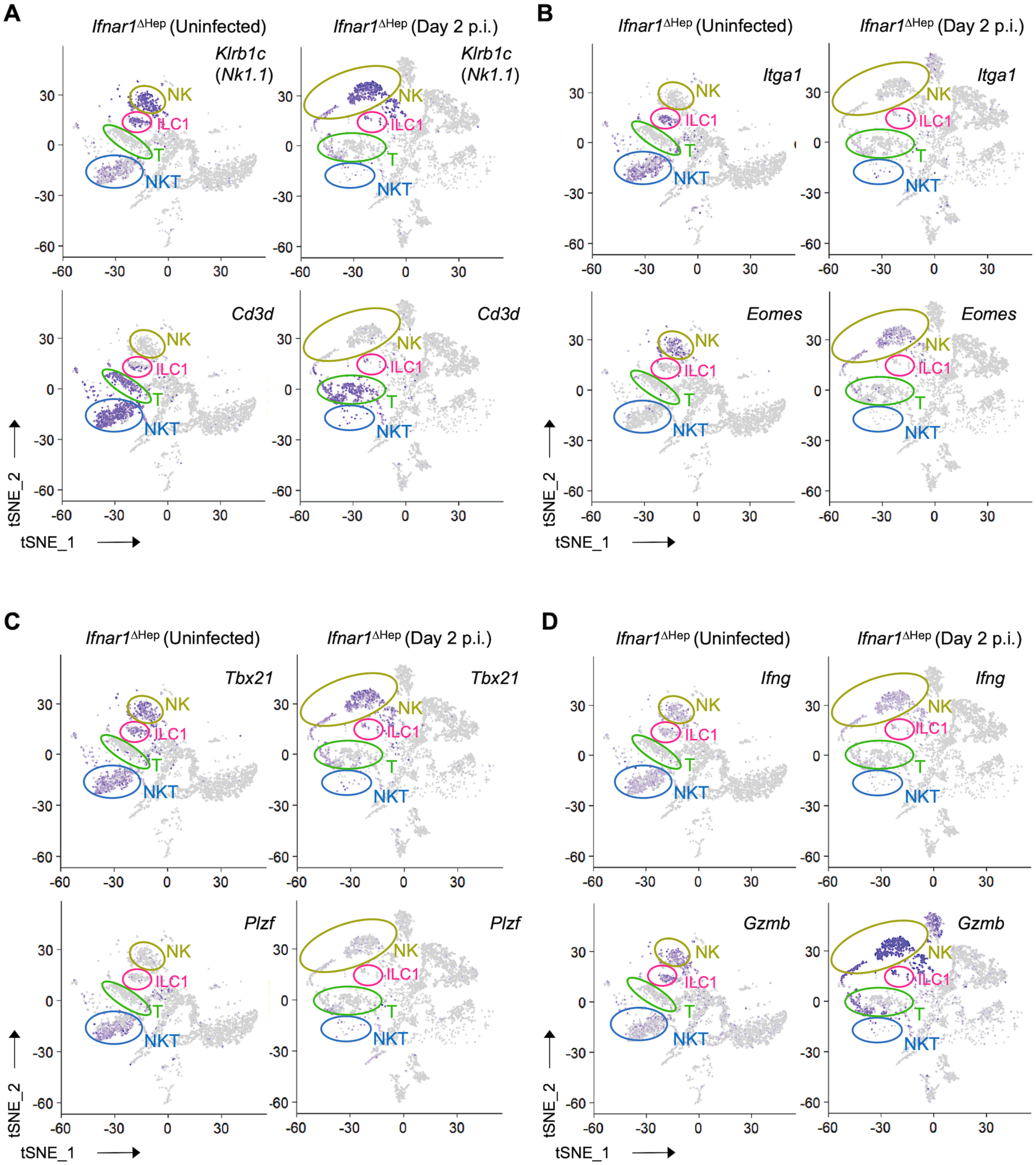

Figure S4. NK1.1+ subsets in the livers of *Ifnar1*$^{\triangle Hep}$ mice before and 2 days after HAV infection. Cohorts of *Ifnar1*$^{\triangle Hep}$ mice were either challenged i.v. with 2×10^7 GE of HM175-mp7 (n=2) or were left unchallenged (n=2). Two days later, livers were harvested and subjected to single-cell RNAseq with the resulting transcriptomic data depicted as tSNE plots, as in Figure 2. NK cells (gold ovals) were distinguished from other NK1.1+ (*Klrb1c*) subsets, including ILC1 cells (red ovals) and NKT cells (blue ovals) and CD3+ T cells (green ovals) based on their differential expression of *Cd3d*, *Itga1*, *Eomes*, *Tbx21*, *Plzf*. Each subset was also analyzed for their expression of *Ifng* and *Gzmb*. **(A)** The top plots show cells expressing *Klrb1c* and the

bottom plots show cells expressing *Cd3d*. **(B)** The top plots show cells expressing *Itga1* and the bottom plots show cells expressing *Eomes*. **(C)** The top plots show cells expressing *Tbx21* (encodes Tbet) and the bottom plots show cells expressing *Plzf* (aka, *Zbtb16*). **(D)** The top plots show cells expressing *Ifng* and the bottom plots show cells expressing *Gzmb*.
