# Supplementary material for: Natural killer cells and IFN-γ protect against liver injury during HAV infection in mice
Source: J Virol. 2025 Sep 19;99(10):e01395-25. doi: 10.1128/jvi.01395-25 (PMC12548451; doi:10.1128/jvi.01395-25)
Supplement: Figure S5 — Immune responses in the livers of Ifnar1ΔHep mice during HAV infection. [file jvi.01395-25-s0005.pdf]

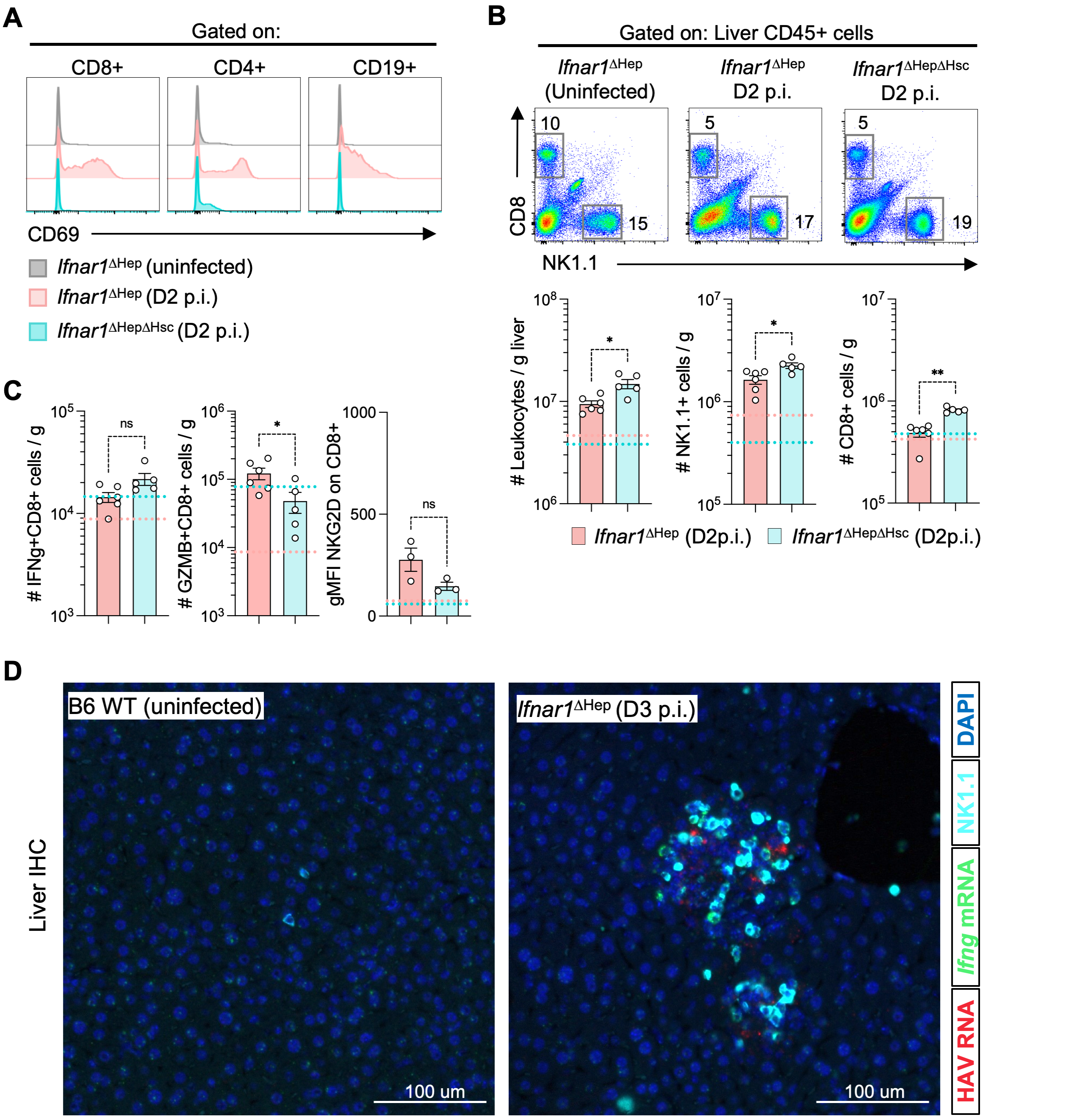

Figure S5. Immune responses in the livers of *Ifnar1*$^{\Delta Hep}$ mice during HAV infection. *Ifnar1*$^{\Delta Hep}$ and *Ifnar1*$^{\Delta Hep\Delta Hsc}$ mice (n= 5-6 / group) were challenged i.v. with 2x10^7 GE of HM175-mp7. At day 2 post-infection, livers were harvested to quantify immune cell populations by flow cytometry and by RNA-scope. **(A)** Representative histograms show the proportion of live intrahepatic T cells and B cells expressing CD69 in uninfected or infected *Ifnar1*$^{\Delta Hep}$ or *Ifnar1*$^{\Delta Hep\Delta Hsc}$ mice. **(B)** Representative FACS plots show the proportion (%) of CD8+ T cells and NK1.1+ cells among liver CD45+ cells. The bar graphs show cell number of CD45+ cells (left), NK1.1+ cells (middle), and CD8+ T cells (right) per gram of liver. **(C)** Bar graphs show the number of IFNγ (left) or granzyme-B (middle) expressing CD8+ T cells per gram of liver. The expression level of NKG2D on CD8+ cells was assessed by gMFI (right). Each symbol

represents an individual mouse. Horizontal dashed lines depict the normal range for naïve *Ifnar1*$^{\Delta Hep}$ (coral) or *Ifnar1*$^{\Delta Hep \Delta Hsc}$ (turquoise) mice. **(D)** Dual immunohistochemical (IHC) and RNA scope staining of HAV RNA (red), *Ifng* mRNA (green), NK1.1 (cyan), and DAPI (blue) in liver sections from an uninfected mouse or *Ifnar1*$^{\Delta Hep}$ mouse at day 3 post-infection (white bar = 100 μm). Significance was assessed by *t*-test with Welch's correction for panel B (left, middle) and panel C; the Mann-Whitney test was used in panel B (right).
