# Supplementary material for: Natural killer cells and IFN-γ protect against liver injury during HAV infection in mice
Source: J Virol. 2025 Sep 19;99(10):e01395-25. doi: 10.1128/jvi.01395-25 (PMC12548451; doi:10.1128/jvi.01395-25)
Supplement: Figure S6 — NK cells limit liver injury during HAV infection. [file jvi.01395-25-s0006.pdf]

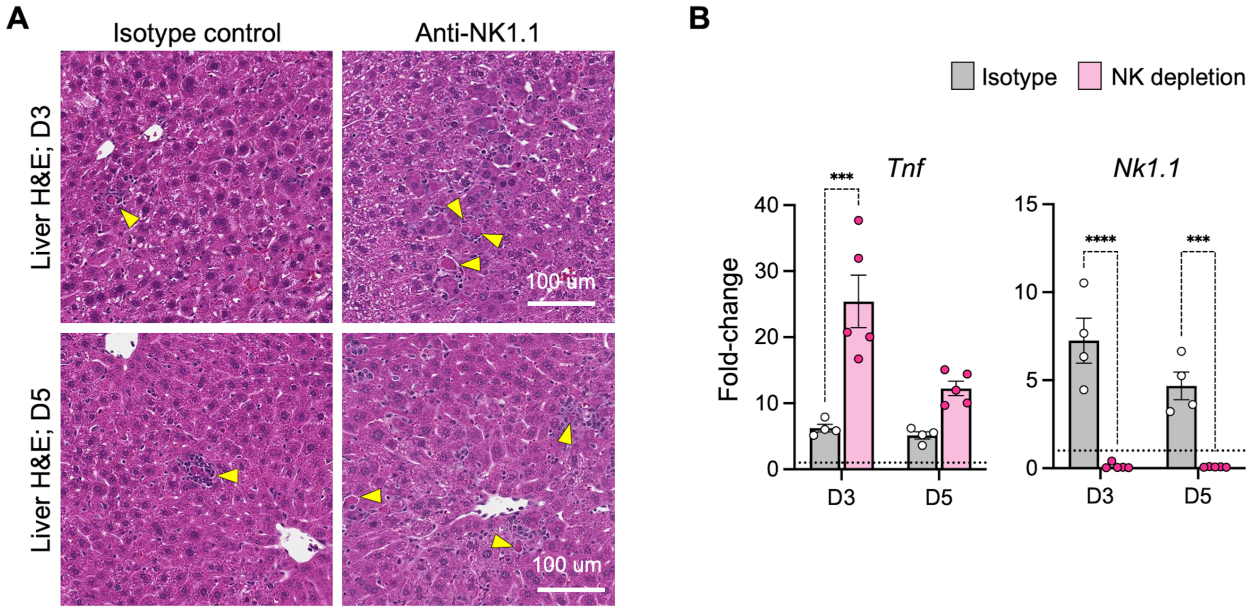

Figure S6. NK cells limit liver injury during HAV infection. NK-replete or NK-depleted *Ifnar1*$^{\Delta Hep}$ mice were challenged with HAV (2x10^7 GE, iv), and their livers were analyzed at days 3 and 5 post-infection for histology and gene expression. **(A)** H&E-stained liver sections with yellow arrows indicating apoptotic hepatocytes (white bar = 100 µm). **(B)** RT-qPCR quantification of liver *Tnf* and *Nk1.1* transcripts, normalized to *β-actin* mRNA. *Nk1.1* is included to verify the loss of NK cells in mice treated with PK136. Gray bars, NK-replete livers; pink bars, NK-depleted livers. Significance was assessed by 2-way ANOVA with Šidák's test for multiple comparisons.
